# The nature of the ligand’s side chain interacting with the S1'-subsite of metallocarboxypeptidase T (from *Thermoactinomyces vulgaris*) determines the geometry of the tetrahedral transition complex

**DOI:** 10.1371/journal.pone.0226636

**Published:** 2019-12-30

**Authors:** Valery Kh. Akparov, Vladimir I. Timofeev, Galina E. Konstantinova, Ilyas G. Khaliullin, Inna P. Kuranova, Tatiana V. Rakitina, Vytas Švedas

**Affiliations:** 1 Protein Chemistry Department, Federal Institution "State Research Institute of Genetics and Selection of Industrial Microorganisms of the National Research Center "Kurchatov Institute", Moscow, Russia; 2 Protein Factory, National Research Centre “Kurchatov Institute”, Moscow, Russia; 3 Laboratory of X-ray analysis methods and synchrotron radiation, Shubnikov Institute of Crystallography of Federal Scientific Research Centre “Crystallography and Photonics” of Russian Academy of Sciences, Moscow, Russia; 4 Kurchatov center of synchrotron-neutron research, National Research Centre “Kurchatov Institute”, Moscow, Russia; 5 Laboratory of ion and molecular physics, Moscow Institute of Physics and Technology, Dolgoprudny, Moscow region, Russia; 6 Laboratory of Hormonal Regulation Proteins, Shemyakin−Ovchinnikov Institute of Bioorganic Chemistry, Russian Academy of Sciences, Moscow, Russia; 7 Faculty of Bioengineering and Bioinformatics, Belozersky Institute of Physicochemical Biology, Lomonosov Moscow State University, Moscow, Russia; Russian Academy of Medical Sciences, RUSSIAN FEDERATION

## Abstract

The carboxypeptidase T (CPT) from *Thermoactinomyces vulgaris* has an active site structure and 3D organization similar to pancreatic carboxypeptidases A and B (CPA and CPB), but differs in broader substrate specificity. The crystal structures of CPT complexes with the transition state analogs N-sulfamoyl-L-leucine and N-sulfamoyl-L-glutamate (SLeu and SGlu) were determined and compared with previously determined structures of CPT complexes with N-sulfamoyl-L-arginine and N-sulfamoyl-L-phenylalanine (SArg and SPhe). The conformations of residues Tyr255 and Glu270, the distances between these residues and the corresponding ligand groups, and the Zn-S gap between the zinc ion and the sulfur atom in the ligand’s sulfamoyl group that simulates a distance between the zinc ion and the tetrahedral sp^3^-hybridized carbon atom of the converted peptide bond, vary depending on the nature of the side chain in the substrate’s C-terminus. The increasing affinity of CPT with the transition state analogs in the order SGlu, SArg, SPhe, SLeu correlates well with a decreasing Zn-S gap in these complexes and the increasing efficiency of CPT-catalyzed hydrolysis of the corresponding tripeptide substrates (ZAAL > ZAAF > ZAAR > ZAAE). Thus, the side chain of the ligand that interacts with the primary specificity pocket of CPT, determines the geometry of the transition complex, the relative orientation of the bond to be cleaved by the catalytic groups of the active site and the catalytic properties of the enzyme. In the case of CPB, the relative orientation of the catalytic amino acid residues, as well as the distance between Glu270 and SArg/SPhe, is much less dependent on the nature of the corresponding side chain of the substrate. The influence of the nature of the substrate side chain on the structural organization of the transition state determines catalytic activity and broad substrate specificity of the carboxypeptidase T.

## Introduction

Metallocarboxypeptidases have attracted increasing attention due to their role in many vital processes including digestion of food proteins, regulation of hormone maturation, cancer, processing of neuropeptides, inflammation, thrombosis, fibrinolysis, etc. [1–3] as well as the growing application of these enzymes in biotechnology. As a result, the mechanisms that govern the catalytic properties of metallocarboxypeptidases, their substrate specificity, and modulation, are of primary interest. However, even in the case of the most intensively studied metallocarboxypeptidase, carboxypeptidase A (CPA), which is one of the first enzymes to be isolated in a pure crystalline form and whose X-ray structure has been solved, the catalytic mechanism is still under continuous discussion [4–9]. It is generally believed that metallocarboxypeptidase catalyzed hydrolysis of peptide substrate is initiated by the attack of the zinc-bonded water molecules on the scissile peptide bond to generate a tetrahedral transition state which is known to be stabilized by the zinc ion and the guanidinium moiety of Arg127. Chemically stable transition state mimics can be used as an effective tool for studying the intimate details of the enzyme catalytic mechanism [10,11].

Substrate recognition and binding to the active site are the best studied steps of metallocarboxypeptidase catalysis, whereas the rate-limiting chemical step involving the conversion of substrates to products and the characterization of the transition states, as well as the factors that determine the substrate profile of each enzyme in this family are not completely understood. It has been documented that CPA and some other metallocarboxypeptidases, including carboxypeptidase T from *Thermoactinomyces vulgaris* (CPT), operate according to the induced fit mechanism. Substrate binding by carboxypeptidases is followed by a remarkable conformational change of the conservative tyrosyl residue in the active site of these enzymes where the phenolic-OH of Tyr248 residue of CPA moves by 12 Å from the surface to the substrate’s terminal carboxyl group of the peptide bond to be hydrolyzed [10], and the hydroxyl group of the corresponding Tyr255 residue in the CPT active site loop Pro248-Asp258 moves more than 10 Å [11]. Substrate-induced conformational changes of Tyr residues are also accompanied by the repositioning of the neighboring residues, whose move is dependent on the substrate’s structure. The induced fit effects, i.e. scale of conformational changes observed in carboxypeptidase catalysis, are large compared to other enzymes.

The role of conformational changes in substrate binding, catalysis and product release has been analyzed for enzymes with available crystal structures for the apo, substrate and product-bound states. The conformational changes of the functional regions involved in catalysis and ligand binding have been determined [12–15]. The authors have found that most enzymes undergo relatively small global conformational changes and particularly small changes in the catalytic residue geometry, usually less than 1 Å. Only in some enzymes there was significant movement of the binding residues, usually on surface loops. Thus, it can be expected that small-scale changes (less than 1 Å) in the position of catalytic residues in the transition states at the conversion of different substrates could be decisive for enzyme’s catalytic activity.

Other factors important for an enzyme’s catalytic activity and substrate specificity can be related to the non-productive binding of a substrate or necessity to reposition the substrate in the course of its catalytic conversion in the active site of the enzyme. In CPT catalysis it has been shown, for example, that the side chain of the positively charged substrate should undergo repositioning when moving from the enzyme-substrate complex to the transition state, whereas the side chain of the hydrophobic substrate does not need repositioning [16]. However, it is not clear how different the structural organization of the transition complexes is at conversion of different substrates by CPT and what factors influence its substrate specificity. As CPT has a quite wide substrate profile, it would be also interesting to understand the differences in the mechanisms governing this property in the homologous enzymes CPA and CPB, whose specificity is significantly limited.

CPA and CPB have narrow substrate specificity and catalyze the cleavage of hydrophobic and positively charged residues, respectively, from the C-termini of the peptides. This is evidenced by the hydrophobic and charge complementarity of their primary specificity pockets and the side chains of the cleaved residue. At the bottom of the primary specificity pocket of CPA isoleucine 255 is present, while the CPB has aspartic acid 255, which interact, respectively, with hydrophobic and positively charged substrates. A distinctive feature of CPT is its wide substrate specificity [17]. CPT is able to remove C-terminal hydrophobic and, less effectively, positively charged residues, despite the presence of the negatively charged residue aspartic acid 260 (numbering according to CPT) at the bottom of the primary specificity pocket and only 2 hydrophobic residues in this pocket versus 4 in the CPA. Furthermore, CPT is also able to cut off negatively charged residues [18]. Replacing all the residues of the primary specificity pocket (S1’-subsite according to Schechter and Berger nomenclature [19] of CPT with the corresponding residues of CPB did not lead to narrowing or shifting of its substrate specificity towards CPB, although the location of the residues lining the primary specificity pocket of such mutant corresponded or was very close to their location in the CPB [20]. These findings indicate that substrate specificity of CPT is regulated by a special mechanism and not just by the substrate recognition due to electrostatic or hydrophobic complementarity of the substrates’ side chains and the S1’-subsite of the active center.

The main goal of this work was to study how the substrate’s side chain interacting with the S1'-subsite of metallocarboxypeptidase T from *Thermoactinomyces vulgaris* influences the geometry of the tetrahedral transition complex and the enzyme’s catalytic activity. To answer this question, the crystal structures of CPT complexes with the transition state analogues of the cleavage reactions of various substrates were obtained, the binding of the transition state analogues in the active center of the enzyme were studied experimentally and quantitatively characterized, the kinetics of CPT-catalyzed conversion of the corresponding substrates were investigated and the kinetic parameters of the enzymatic reactions were determined.

## Materials and methods

### Materials

Subtilisin 72 [21], peptide substrates ZAAL-pNA (N-benzyloxycarbonyl-alanyl-alanyl-leucine p-nitroanilide) [22], ZAAR (N-benzyloxycarbonyl-alanyl-alanyl-arginine) [23], and ZAAL (N-benzyloxycarbonyl-alanyl-alanyl-leucine) [24] were prepared in our laboratory. Affinity sorbent ([N-(ε-amino-caproyl)-p-aminobenzyl]succinyl-Sepharose 4B (CABS-Sepharose) was prepared according to previously described protocols [25]. Ultrafiltration cells and PM10 membranes were from Amicon (Millipore-Sigma, Birlington, MA), glass capillaries 60-mm in length and inner diameter of 0.5 mm for CPT crystallization from Confocal Science Inc. (Tokyo, Japan). Deionized water with resistance of 19 MOhm/cm was prepared on the MilliQ system (Merck Millipore, Middlesex Turnpike Billerica, MA), 2-methyl-2,4-pentadiol (MPD), L-cystine and L-cysteine, diisopropyl fluorophosphate, isopropyl-β-D-thiogalactoside (IPTG), 3-[(3-cholamidopropyl)dimethylammonio]-1-propanesulfonate (CHAPS), and 4-morpholinoethansulfonic acid (MES), and porcine carboxypeptidase B were obtained from Sigma-Aldrich (St. Louis, MO). The kinetic experiments were performed in the thermostated cell of the Shimadzu UV-1800 spectrophotometer (Shimadzu, Kyoto, Japan) connected with IBM PC.

### Expression of the CPT proenzyme in E. coli as inclusion bodies and subsequent in vitro renaturation of the enzyme

The *pro-cpT* gene from *Thermoactinomyces vulgaris* was expressed in *E*. *coli* BL21(DE3)pLysS cells according to instructions of the producer Novagen (Merck Bioscience AG, Leufelfingen, Switzerland) [26]. Following transformation, bacteria were inoculated in test-tubes containing 5 mL of LB media with 0.12 mg/mL ampicillin plus 0.034 mg/mL chloramphenicol and incubated for 18 h at 28°C. After that the cells were inoculated in flasks with LB media in the presence of 1 mM IPTG, 0.12 mg/mL ampicillin and 0.034 mg/mL chloramphenicol. Culture was grown for 6 h at 28°C. After IPTG-induced expression, bacteria were precipitated and sonicated.

Native CPT was isolated from inclusion bodies as described in [27]. The inclusion bodies were separated by centrifugation, washed with 0.05% CHAPS (w/v), 2 M NaCl and water, and dissolved in 8 M urea up to the final concentration of 5 mg/mL. Then the protein solution was rapidly diluted 10-fold with 50 mM Tris-HCl/30% glycerol (v/v, hereinafter the percentage is in a volume-to-volume ratio)/0.5 M NaCl/10 mM CaCl_2_, pH 8.0, and incubated for 16 h at 37°C. The solution was further diluted 2-fold with 50 mM Tris- HCl/0.5 M NaCl/10 mM CaCl_2_ buffer with pH 8.0, and concentrated by ultrafiltration up to the volume of 20 mL. In order to activate the proenzyme, subtilisin 72 was added at the molar ratio 1/200 CPT/subtilisin and the solution was incubated for 4 h at 37°C. Then subtilisin was inactivated by the addition of diisopropyl fluorophosphate. Thereafter, the protein solution was concentrated by ultrafiltration up to the volume of 0.5 mL.

### Purification of CPT

After concentration, the protein was acidified up to pH 6.0 using 100 mM MES buffer pH 5.8. The protein-containing solution was then loaded on a CABS Sepharose (20 mL) column equilibrated with 10 mM MES, pH 6.0, containing 0.5 M NaCl, 10 mM CaCl_2_ and 0.1 mM ZnSO_4_. CPT was eluted with 10 mM Tris-HCl/0.5 M NaCl/10 mM CaCl_2_ buffer pH 8.0. Fractions containing the active protein were pooled, concentrated to 1 mL, and the buffer was replaced by the crystallization buffer (0.01 M MES / NaOH, pH 6.0, containing 1 mM CaCl_2_, 0.1 mM ZnSO_4_ and 0.25 M NaCl) by three rounds of ultrafiltrations using an Amicon cell with 10-fold dilution. Finally, the protein was concentrated to 10 mg/mL and filtered aseptically using Centripack centrifuge holders. The solution was used for protein crystallization. The absence of subtilisin activity was confirmed using the specific ZAAL-pNA chromogenic substrate.

### Protein concentration

Protein concentration was measured by the Bradford method [28]. SDS-PAGE was performed as previously described by Laemmli [29].

### Study of enzymatic properties of the CPT

The subtilisin activity in the CPT solution was assayed with the specific substrate ZAAL-pNA in a concentration of 0.1 mM in 50 mM Tris-HCl/10 mM CaCl_2_ buffer pH 8.5. The reaction mixture was incubated at 37°C until the pale-yellow color appeared. The reaction was stopped by the addition of 0.5 M HCl. The enzyme amount was calculated taking into account time-to-color measurement and that the molar absorption coefficient of *p*-nitroaniline at 410 nm equals to 8200 M^-1^cm^-1^ [22], and the activity of pure enzyme is 10 units/mg. The enzymatic activity of CPT was characterized by its ability to chip off an arginine residue from ZAAR, glutamic acid residue from ZAAE and leucine residue from ZAAL. Hydrolysis of ZAAL, ZAAE and ZAAR was performed at 25°C in 250 mM Tris-HCl/10 mM CaCl_2_ buffer pH 7.5, containing 4–400 mM ZAAL, 8–800 mM of ZAAE or 6–600 mM ZAAR. The observed absorption decrease at 225 nm was recalculated to reaction rate using the peptide bond molar absorption coefficient ε_225_ 376 M^-1^cm^-1^ (peptide bond absorption). The enzyme concentration was chosen individually for each mutant. The initial rates at 6–7 substrate concentrations from the above range were determined. The kinetic data were processed by nonlinear fitting to Michaelis-Menten equation in SciDAVis (http://scidavis.sourceforge.net/index.html) to obtain k_cat_ and K_M_.

### Crystal growth

Crystals of CPT complexes with SLeu and SGlu were grown in microgravity in a capillary using the counter-diffusion technique. Equipment and technology developed by the Aerospace Agency of Japan JAXA were used for crystal growth, as described by S. Takahashi, *et al*., and I.P. Kuranova, *et al*. [30, 31]. The protein concentration was 10–12 mg/mL. Protein was dissolved in 10 mM MES buffer pH 6.0, containing 0.25 M NaCl, 0.1 mM ZnSO_4_, and 1 mM CaCl_2_. The precipitating solution contained 1.6 M (NH_4_)_2_SO_4_, 0.1 mM ZnSO_4_, 1 mM CaCl_2_ and 5% MPD in 50 mM MES buffer pH 6.0, and an appropriate ligand (100 mM).

A glass capillary (0.5 mm in diameter) with the protein-containing solution served as a crystallization device. One end of the capillary was hermetically sealed; a silicon pipe (0.5 mm) filled with 1% agarose gel and plunged into a cylinder with the precipitating solution was connected to the other end. A few crystallization devices placed in special boxes were delivered to the International Space Station, where crystals were grown in a thermostat at 20°C. Slow diffusion of the precipitant into the protein solution through a gel layer under zero gravity conditions improves diffraction properties of crystals [32]. To collect diffraction data using a synchrotron source, the crystal was taken off the capillary and placed first to the precipitant solution, then to cryosolution and frozen in a nitrogen vapor flow. Along with the precipitant components, the cryosolution also contained 20% glycerol (v/v).

### Collection and processing of diffraction data

Diffraction sets of the grown crystals were collected on the Spring 8 (Japan) synchrotron at the temperature of 100K at the BL41XU station. The PILATUS device was used as a detector. The diffraction data were obtained using a single crystal by rotation. The wavelength was 0.8 Å; the distance from crystal to detector was 200 mm and the oscillation angle was 0.1° for both CPT+SLeu and CPT+SGlu complexes respectively; the angle of rotation was 360°. The processing of the sets of experimental intensities was performed using the HKL-2000 software [33]. The sets were processed to the resolution of 1.93 Å for CPT+SGlu and 1.90 Å for CPT+SLeu. The crystals belonged to space group P6_3_22. The independent part of the unit cell contains one molecule of the enzyme. The statistic characteristics of the sets are presented in Table 1.

**Table 1:** Statistical characteristics of experimental data set and refinement of the structure of the CPT–ligand complexes.

Data collection		
Space group	P6_3_22	P6_3_22
Cell parameters	a = b = 157.8, c = 104.8 Å; α = β = 90° γ = 120°	a = b = 158.7, c = 105.6 Å; α = β = 90° γ = 120°
Resolution (Å)	30.0–1.9 (2.0–1.9)	30.0–1.93 (2.03–1.93)
Unique reflections	60665	58550
Completeness (%)	99.86	98.98
I/σ(I)	14.16 (2.04)	14.75 (2.38)
Rmrgd-F (%)	13.0 (35)	13.7 (31)
Refinement statistics		
PDB entry	6GO2	6SN6
Resolution range (Å)	30.0–1.9 (1.9–1.949)	30.0–1.93 (1.98–1.93)
Unique reflections	57684	55566
Rcryst	0.141	0.112
Rfree (%)	0.163	0.144
No. of protein atoms in final model	2581	2678
Mean B value	14.38	17.73
RMS		
Bonds (Å)	0.008	0.020
Angles (°)	1.357	1.951
Ramachandran plot		
Most favoured (%)	89.2%	91.6%
Allowed (%)	10.4%	8.0%
Disallowed	0.4%	0.4%

### Structure determination and refinement

The structures of CPT+SLeu (PDB ID: 6GO2) and CPT+SGlu (PDB ID: 6SN6) complexes were determined by the molecular replacement method using the Phaser software [34] and CPT atomic coordinates (PDB ID: 3QNV [35]) as a starting model. For structure refinement, the Refmac program [36] was used. The manual correction of the models was performed using Coot software [37], and electron density maps were calculated with 2|Fo|–|Fc| and |Fo|–|Fc| coefficients. Water molecules and calcium ions were located on the electron density maps, and difference Fourier synthesis revealed electron density in the active site, which was identified as a ligand. Ligands were refined with occupation of 100%. The characteristics of the refined structures are presented in Table 1.

### Kinetic parameter determination

The kinetic parameters of ZAAL, ZAAE and ZAAR hydrolysis by CPT were determined as described earlier [16].

### Determination of inhibition constants

Integral kinetic curves of the tripeptide substrates hydrolysis catalyzed by CPT in the presence and absence of the inhibitors were analyzed in coordinates:

$\frac{t}{\ln(s_{0}/s)}$ versus $\frac{s_{0}-s}{\ln(s_{0}/s)}$, [38], where t is time, s and s_0_ –running and initial substrate concentrations. This allowed the calculation of the apparent (K_M_/V_max_)_app_ value at each used concentration of the inhibitor (I). To determine the inhibition constant (K_I_), the dependence of the calculated (K_M_/V_max_)_app_ parameter on the inhibitor concentration was examined in accordance with the linear equation:

$$\left(\frac{K_{M}}{V_{\max}}{)}_{app}=(\frac{K_{M}}{V_{\max}}{)}_{0}(1+\frac{I}{K_{I}}\right)$$

### Visualization

Structure superposition was carried out on all alpha-carbon atoms.

### Synthesis of transition-state analogs

#### Dibenzyl *N*-({[(benzyloxy)carbonyl]amino}sulfonyl)-L-glutamate. MW = 540.59

Benzyl alcohol (1.2 mL, 11.5 mmol) was added slowly at 0°C to the stirring chlorosulfonyl isocyanate (1 mL, 11.5 mmol) solution in anhydrous dichloromethane (10 mL), and the stirring was continued for 30 min at 0°C. N,N-Diisopropylethylamine (6 mL, 34 mmol) in anhydrous dichloromethane was added to the solution. The resulting mixture was then added dropwise to an ice-chilled solution of L-glutamic acid dibenzyl ester *p*-toluenesulfonate (6.0 g, 12 mmol) and N,N-diisopropylethylamine (2.5 mL, 14.3 mmol) in anhydrous dichloromethane (25 mL). The obtained solution was stirred for 2 h at 20-22ºC and evaporated under reduced pressure. The residue was dissolved in a mixture of ethyl acetate (50 mL) and water (50 mL), the ethyl acetate layer was washed with 5% H_2_SO_4_ solution (50 mL x 2) and brine (50 mL x 2) and dried over anhydrous Na_2_SO_4_. The ethyl acetate solution was filtered from Na_2_SO_4_ and concentrated in vacuo to afford an oily residue. The crude product was purified by flash chromatography on silica gel (CHCl_3_/toluene/AcOEt gradient 9: 9: 2 to 9: 9: 4) to give the oily product. The oil became crystalline overnight at 4°C. The crystals were washed with petroleum ether 40–70 (50 mL x 2) and dried in vacuo. Yield: 3.087 g, 5.71 mmol, 49.6%.

TLC AcOEt / n-hexane (2: 3) R_f_ 0.50, CHCl_3_/toluene/AcOEt (3: 3: 1) R_f_ 0.35; mp 75,2–76,7°C; [α] ^25^_D_ +1,4 (*c* 1.5 MeOH); MS [M+H^+^] 541.15; ^1^H NMR (DMSO-*d*_6_, 400 mHz) δ 11.43 (s, 1H NH), 8.61 (d, 8.58 Hz 1H NH), 7.34–7.36 (m, 15H CH_2_Ph), 5.03–5.12 (m, 6H CH_2_Ph), 4,08–4,14 (dt, 8.58 Hz, 4.77 Hz 1H CH), 2.43–2.47 (m, 2H CH_2_), 1.77–2.06 (m, 2H CH_2_); Anal. (C_27_H_28_N_2_O_8_S) C, H, N.

#### N-(aminosulfonyl)-L-glutamic acid

**MW = 226.21.** Dibenzyl *N*-({[(benzyloxy)carbonyl]amino}sulfonyl)-L-glutamate (3.03 g, 5.605 mmol) was dissolved in a mixture of methanol (60 mL) and cyclohexene (10 mL, ~100 mmol). A suspension of 10% Pd on carbon (1 g) in a mixture of 95% ethanol (6 mL) and water (3 mL) was added to the solution. The reaction mixture was stirred for 1 h at 55°C with the reflux solvents. The reaction mixture was then cooled down to room temperature and filtered from Pd on carbon. The Pd on carbon was washed with methanol (30 mL x 2). The collected solution was evaporated under reduced pressure. The oily residue was dissolved in ethyl acetate (30 mL) and evaporated under reduced pressure. The oil became partly crystalline within 36 h at 4°C. The solid was treated with diethyl ether (30 mL) to give crystalline precipitation. The crystals were filtered and washed with diethyl ether (30 mL x 2), petroleum ether 40–70 (30 mL) and dried in vacuum. Yield: 1.203 g, 5.318 mmol, 94.9%.

TLC n-BuOH / AcOH / H_2_O (4: 1: 1) R_f_ 0.74; CHCl_3_ / MeOH / AcOH / H_2_O (50: 15: 5: 2) R_f_ 0.36; mp 124 (decomposing); [α] ^25^_D_ -12,0 (*c* 1.0 MeOH); MS [M+H^+^] 227.02; ^1^H NMR (DMSO-*d*_6_, 400 mHz) δ 12.41 (s, 2H OH), 8.96 (d, 6.04 Hz 1H NH), 6.56 (s, 2H NH), 3.78 (q, 1H 4.14 Hz CH), 2.33 (m, 2H CH_2_), 1.69–1.99 (m, 2H CH_2_); Anal. (C_5_H_10_N_2_O_6_S) C, H, N.

#### Benzyl N-({[(benzyloxy)carbonyl]amino}sulfonyl)-L-leucine

**MW = 434.5.** Benzyl alcohol (1.2 mL, 11.5 mmol) was added slowly at 0°C to the stirring chlorosulfonyl isocyanate (1 mL, 11.5 mmol) solution in anhydrous dichloromethane (10 mL), and the stirring was continued for 30 min at 0°C. Triethylamine (4.73 mL, 34 mmol) in anhydrous dichloromethane was added to the solution. The resulting mixture was then added dropwise to an ice-chilled solution of L-leucine benzyl ester p-toluenesulfonate (5.0 g, 12.7 mmol) and N,N-diisopropylethylamine (2.5 mL, 14.3 mmol) in anhydrous dichloromethane (25 mL). The obtained solution was stirred overnight at room temperature and evaporated under reduced pressure. The residue was dissolved in a mixture of ethyl acetate (50 mL) and water (50 mL), the ethyl acetate layer was washed successively with 2.5% H_2_SO_4_ solution (50 mL x 2) and brine (50 mL x 2) and dried over anhydrous Na_2_SO_4_. The ethyl acetate solution was filtered from Na_2_SO_4_ and concentrated in vacuum to afford an oily residue. The oil became crystalline overnight at 4°C. The crystals were washed with hexane (50 mL x 2) and dried in vacuum.

Yield: 4.252 g, 9.787 mmol, 85.1%.

TLC Toluene-Acetone-AcOH (100: 50: 1), Rf 0.47

1H NMR (400 MHz, DMSO-d6) δ ppm: 11.40 (s, 1 H); 8.56 (d, J = 8.6 Hz, 1 H) 7.49–7.25 (m, 10 H); 5.05-5-16 (m, 4 H); 3.99 (dd, J = 7.5, 6.9 Hz, 1 H); 1.62–1.4 (m, 3 H); 0.8 (dd, J = 6.5, 0.2 Hz, 6 H).

#### N-(aminosulfonyl)-L-leucine sodium salt

**MW = 232.2.** Benzyl N-({[(benzyloxy)carbonyl]amino}sulfonyl)-L-leucine (4.15 g, 9.551 mmol) was dissolved in a mixture of methanol (70 mL) and cyclohexene (10 mL, ~100 mmol). A suspension of 10% Pd on carbon (0.5 g) in a mixture of 95% ethanol (4 mL) and water (2 mL) was added to the solution. The reaction mixture was stirred overnight at room temperature in a hydrogen atmosphere. The reaction mixture was filtered from Pd on carbon. The Pd on carbon was washed with methanol (30 mL x 2). The collected solution was evaporated under reduced pressure to give an oily residue. The crude product was purified by flash chromatography on silica gel using eluent mixture (chloroform–methanol–acetic acid–water 60:15:5:2). The solution after chromatography was evaporated to give an oily product. The oil was dissolved in a mixture of isopropanol (30 mL) and methanol (30 ml) and 1 M NaOH in methanol (6 mL; 6 mmol was added. The solution was evaporated to give an oily product. The oil was treated with ethylacetate to give a solid material. The crystals were filtered and washed with diethyl ether (30 mL x 2), petroleum ether 40–70 (30 mL) and dried in vacuum. Yield: 1.980 g, 8.529 mmol, 89.3%.

TLC chloroform–methanol–acetic acid–water (50:15:5:2) Rf 0.44. n-butanol–acetic acid–water (4:1:1), Rf 0.70.

1H NMR (400 MHz, DMSO-d6) δ ppm: 6.44 (s, 2H); 5.99 (d, J = 6.1 Hz, 1H); 3.47 (dd, J = 10.1, 4.1 Hz, 1 H); 1.86–1.74 (m, 1H); 1.54–1.44 (m, 1H); 1.41–1.31 (m, 1 H); 0.84 (dt, J = 6.5 Hz, 6 H)

#### Z-Ala-Ala-Glu-OH HCl, H-Ala-Glu(OMe)-OMe

**MW = 282.7.** Boc-Ala-OPfp—pentafluorophenyl ester of Boc-Alanine, (5.33 g, 15.0 mmol) was dissolved in N,N-dimethylformamide (50 mL). HCl, H-Glu(OMe)-OMe (3.81 g, 18.0 mmol) was added to the solution. The reaction mixture was cooled down to 0°C in an ice bath and stirred. N,N-Diisopropylethylamine (3.5 mL, 20.0 mmol) was added to the solution dropwise within 30 min. The reaction mixture was stirred overnight at room temperature. Then, the solution was evaporated under reduced pressure. The residue was dissolved in a mixture of ethyl acetate (120 mL) and 2.5% H_2_SO_4_ water solution (120 mL). The ethyl acetate layer was separated and washed successively with 10% KHSO_4_ solution (100 mL), 20% Na_2_SO_4_ solution (100 mL), 5% NaHCO_3_ solution (100 mL x 2) and brine (100 mL) and dried over anhydrous Na_2_SO_4_ for 1 h. The ethyl acetate solution was filtered from Na_2_SO_4_ and concentrated in vacuo to afford an oily residue. The oil was dissolved in ethyl acetate (200 mL) and silica gel (70–230 mesh, 50 g) was added to the solution. The mixture was stirred for 5 min and the solution was filtered off from the silica. The silica was washed with ethylacetate (100 mL x 3) and the combined ethylacetate solution was evaporated. (TLC toluene–acetone–acetic acid (100:50:1) Rf 0.45). The oily product Boc-Ala-Glu(OMe)-OMe was dissolved in dioxane (20 mL) and 4N HCl/dioxane solution was added (50 mL). The solution was stirred to become homogeneous and left for 30 min. Then, the solution was evaporated to the viscous oil. The oily residue was treated with diethyl ether (70 mL) to become solid. The mixture was left overnight in the fridge (4°C). The solid powder was filtered and washed with diethyl ether (50 mL) and hexane (50 mL) and dried in vacuum.

Yield: 3.42 g, 12.09 mmol, 80.6% (based on Boc-Ala-OPfp)

TLC chloroform–methanol–acetic acid–water (60:15:5:2), Rf 0.23

n-butanol–acetic acid–water (4:1:) Rf 0.62

1H NMR (400 MHz, DMSO-d6) δ ppm: 9.07 (d, J = 7.7 Hz, 1H); 8.45–8.21 (br. s, 3H); 4.38 (q, J = 6.7 Hz, 1H); 3.95 (q, J = 7.0 Hz, 1H); 3.61 (s, 3H); 3.50 (s, 3H); 2.28 (qd, J = 16.7, 6.2 Hz, 2H); 1,98–1.90 (m, 2H), 1,85–1,77 (m, 2H); 1.20 (d, J = 7.3 Hz, 6H)

#### Z-Ala-Ala-Glu(OMe)-OMe

**MW = 451.4.** Z-Ala-OPfp—pentaflourophenyl ester of Z-Alanine, (6.0 g, 15.40 mmol) and HCl, H-Ala-Glu(OMe)-OMe (3.35 g, 11.85 mmol) were dissolved in N,N-dimethylformamide (50 mL). The solution was cooled down to 0°C in an ice bath and N,N-diisopropylethylamine (2.8 mL, 16.0 mmol) was added to the solution dropwise within 30 min. The reaction mixture was stirred overnight at room temperature. Then N,N-dimethylethylenediamine (0.5 mL, 5 mmol) was added to the solution and the reaction mixture was evaporated under reduced pressure. The oily residue was dissolved in ethyl acetate (150 mL) and water (150 mL). The organic layer was separated and washed successively with 2.5% H_2_SO_4_ water solution (100 mL x 2), 20% Na_2_SO_4_ solution (100 mL), 5% NaHCO_3_ solution (100 mL x 2) and brine (100 mL) and dried over anhydrous Na_2_SO_4_ for 1 h. The ethyl acetate solution was filtered from Na_2_SO_4_ and concentrated in vacuo to afford an oily residue. The oil of Z-Ala-Ala-Glu(OMe)-OMe became solid overnight at room temperature. The solid material was treated with hexane (50 mL). The powder was filtered off, washed with hexane (50 mL) and dried in vacuum.

Yield: 4.41 g, 9.765 mmol, 82.4%.

TLC chloroform–methanol–acetic acid (45:5:1) Rf 0.45

1H NMR (400 MHz, DMSO-d6) δ ppm: 8.05 (d, J = 7.6 Hz, 1H); 7.98 (d, J = 7.7 Hz, 1H); 7.47 (d, J = 7.6 Hz, 1H); 7.42–7.31 (m, 5H); 5.02 (d, J = 6.4 Hz, 2H); 4.29 (q, J = 6.6 Hz, 1H); 4.21 (q, J = 7.1 Hz, 1H); 4.04 (q, J = 7.0 Hz, 1H); 3.64 (s, 3H); 3.52 (s, 3H); 2.28 (qd, J = 16.7, 6.2 Hz, 2H); 1,98–1.90 (m, 2H), 1,85–1,77 (m, 2H); 1.20 (d, J = 7.3 Hz, 6H)

#### Z-Ala-Ala-Glu-OH

**MW = 423.4.** Z-Ala-Ala-Glu(OMe)-OMe (4.34 g, 9.6 mmol) was dissolved in methanol (100 mL) and evaporated to viscous oil. The residue was dissolved in a mixture of methanol (20 mL) and tetrahydrofuran (20 mL). 1 M NaOH (25 mL; 25 mmol) was added to the solution at 0°C (ice bath). The reaction mixture was stirred for 2 h at 0°C and for 2 h at room temperature. The solution was acidified up to pH 7–8 with 25% H_2_SO_4_ dropwise and evaporated. The residue was dissolved in a mixture of n-butanol (100 mL) and 10% KHSO_4_ solution (100 mL). The organic layer was separated and dried with anhydrous Na_2_SO_4_. The butanol solution was filtered from Na_2_SO_4_ and concentrated in vacuo to produce an oily residue. The oil was treated with diethyl ether (70 mL) to give a white powder. The powder was filtered off, washed with hexane (50 mL) and dried in vacuum.

Yield: 3.041 g, 9.072 mmol, 94.5%.

TLC chloroform–methanol–acetic acid–water (75:15: 5:2) Rf 0.47.

1H NMR (400 MHz, DMSO-d6) δ ppm: 12.70–12.00 (br. s, 2H); 8.05 (d, J = 7.6 Hz, 1H); 7.98 (d, J = 7.7 Hz, 1H); 7.47 (d, J = 7.6 Hz, 1H); 7.42–7.31 (m, 5H); 5.02 (d, J = 6.4 Hz, 2H); 4.29 (q, J = 6.6 Hz, 1H); 4.21 (q, J = 7.1 Hz, 1H); 4.04 (q, J = 7.0 Hz, 1H); 2.28 (qd, J = 16.7, 6.2 Hz, 2H); 1,98–1.90 (m, 2H), 1,85–1,77 (m, 2H); 1.20 (d, J = 7.3 Hz, 6H)

## Results and discussion

### Structure overview

The structures of the CPT complexes with transition state analogs carrying hydrophobic and negatively charged side chains SLeu and SGlu were determined at resolutions of 1.90 and 1.93 Å, respectively. The ligands were refined with occupancies of 100%.

The spatial structures of these complexes generally correspond to the structures of previously obtained complexes of N-sulfamoyl phenylalanine (CPA+SPhe) [39] and N-sulfamoyl arginine (CPT+SArg, CPB+SArg) [40, 11] with carboxypeptidases A, B and T. The tetrahedral sulfamoyl group, that mimics the tetrahedral sp^3^-hybridized carbon atom of the convertible bond, interacts with the residues of the catalytic site of CPT, including the zinc ion coordinated by the side chain functional groups of Glu72, His204, His69, the catalytic residue Glu277, which activates the attacking water molecule, and Arg129, which polarizes the carbonyl of the bond to be cleaved. The sulfamoyl group of the ligand is located asymmetrically with respect to the Zn^2+^ ion. The side chains of Asn146, Arg147, Tyr255 coordinate the C-terminal carboxyl group common to all substrates.

### Structure analysis

**Fig 1:**
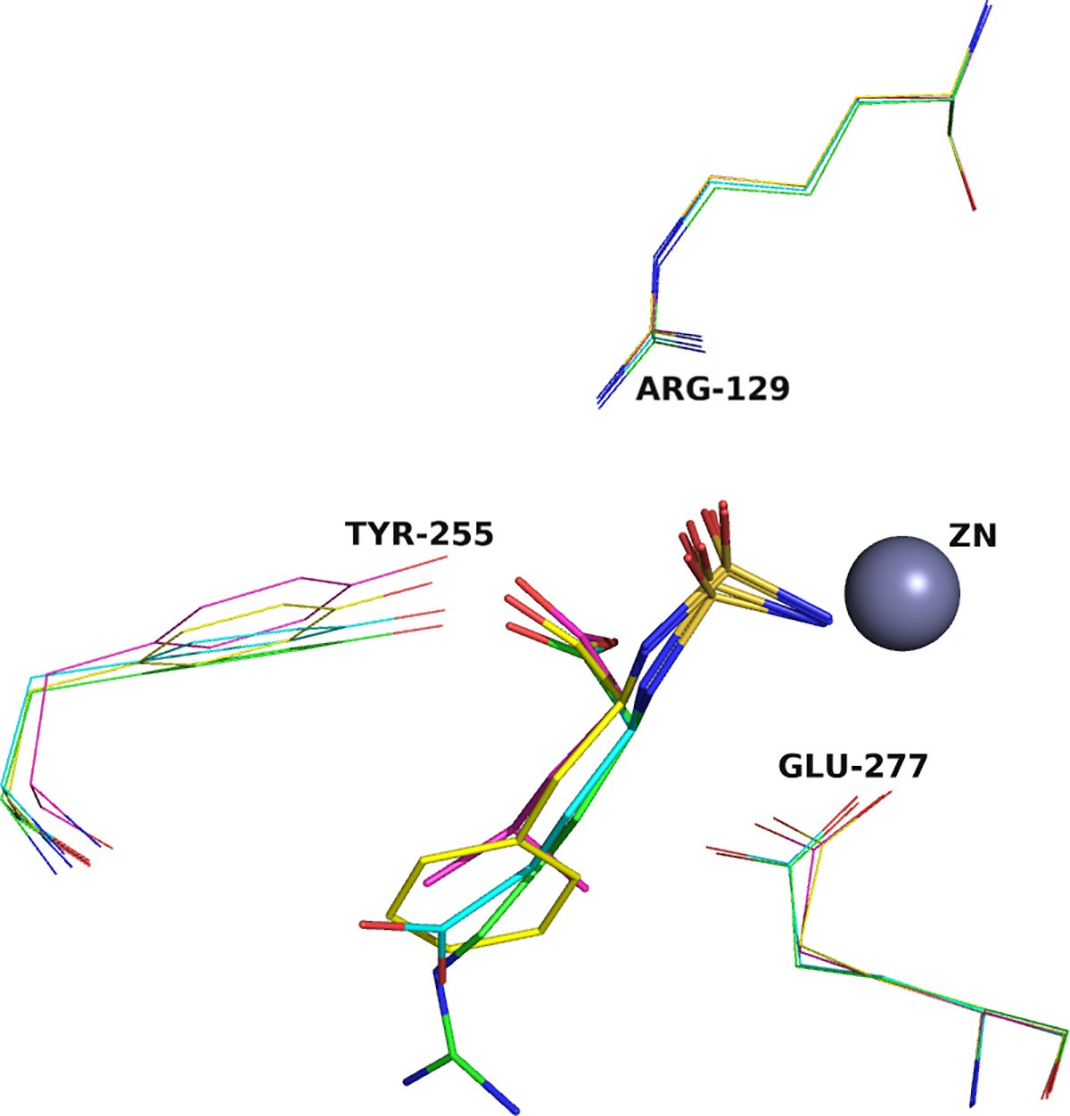
The nature of the inhibitor's side chain influences on the conformation of the transition complex of CPT. Superposition of CPT complexes with SArg (green), SPhe (yellow), SLeu (cyan), SGlu (magenta) is shown. Zinc ion is represented as the gray sphere, amino acid residues of the catalytic site of the CPT's active center are shown by wire models.

The detailed structural comparison of the CPT complexes with transition state analogs carrying side chains of a particular nature—SArg, SGlu, SPhe and SLeu—superimposed on Cα atoms (Fig 1) reveals remarkable differences in the disposition of ligands in the active site of the enzyme. Interestingly, this is related to the tetrahedral sulfur atom and neighboring atoms.

The gap between sulfur atoms in superimposed complexes CPT+SPhe and CPT+SGlu is 0.38 Å, the distance between the N17 nitrogen atoms is 0.76 Å and between the O20 oxygen atoms of the sulfamide groups is 0.65 Å whereas RMSD of the all atoms of these complexes is 0.077Å only (Table 2).

**Table 2:** Distances between Glu277, Tyr255 residues and N-sulfamoyl ligands atoms in superimposed CPT+SGlu and CPT+SArg, CPT+SPhe, CPT+SLeu complexes.

Atom	SGlu—SArg, Å	SGlu—SPhe, Å	SGlu—SLeu, Å
S18	0.18	0.38	0.31
N17	0.14	0.76	0.62
N19	0.12	0.23	0.19
O20	0.26	0.65	0.57
O21	0.21	0.29	0.35
C14	0.08	0.42	0.35
O16	0.33	0.31	0.42
O15	0.04	0.34	0.21
C13	0.03	0.76	0.61
Tyr 255, OH	0.21	0.44	0.75
Glu277, CD	0.05	0.54	0.39
Glu277, OE1	0.12	0.50	0.35
Glu277, OE2	0.17	1.05	0.77
RMSD	0.063	0.077	0.056

The C-terminal carboxyl groups of the ligands are positioned differently in the superimposed CPT complexes with sulfamoyl inhibitors as well as the side chains of the amino acid residues forming the enzyme’s active site. The maximum shift is between Glu277 OE2 in SGlu and SPhe complexes (1.05 Å) that is again much more than RMSD of the atoms in the SArg, SPhe, SLeu complexes and the corresponding atoms in SGlu complex (Table 2).

The distances between the residues of the catalytic subsite and the sulfamoyl group of the ligands also depend strongly on the nature of the side chain of the bound ligand, the maximum difference in these distances is 0.52 Å (O20 SPhe—OE1 Glu277) (Table 3).

**Table 3:** Distances between CPT catalytic subsite residues and N-sulfamoyl ligands.

No	Ligand atom	Distance, [Å]				CPT residues
		SGlu	SArg	SPhe	SLeu	
1	[O16]	3.59	3.75	3.61	3.64	ARG 129[NH2]
2	[O15]	3.54	3.51	3.69	3.35	ARG 129[NH2]
3	[O21]	2.66	2.78	2.62	2.68	ARG 129[NH1]
4	[O21]	3.16	3.18	3.28	3.29	ARG 129[NH2]
5	[O15]	2.91	2.92	3.04	3.01	ASN 146[ND2]
6	[O15]	2.80	2.80	2.94	2.81	ARG 147[NH1]
7	[O16]	2.93	2.82	2.76	2.83	ARG 147[NH2]
8	[N19]	3.12	3.13	2.96	2.97	THR 205[O]
9	[N17]	3.08	3.14	3.13	3.09	TYR 255[OH]
10	[O16]	2.68	2.71	2.66	2.64(O15)	TYR 255[OH]
11	[N19]	3.21	3.26	3.30	3.21	GLU 277[OE1]
12	[N17]	2.71	2.71	3.10	2.9	GLU 277[OE2]
13	[O20]	4.03	4.08	4.57	4.46	GLU 277[OE1]
14	[S18]	3.20	3.18	3.10	3.08	Zn^2+^
15	[O20]	4.20	4.28	4.10	4.04	Zn^2+^
16	[O21]	3.21	3.31	3.10	3.02	Zn^2+^

The maximum difference in the lengths of the hydrogen bond between the ligand and the catalytic groups of the enzyme (the distance [N17]—GLU 277 [OE2]) is 0.39 Å.

**Fig 2:**
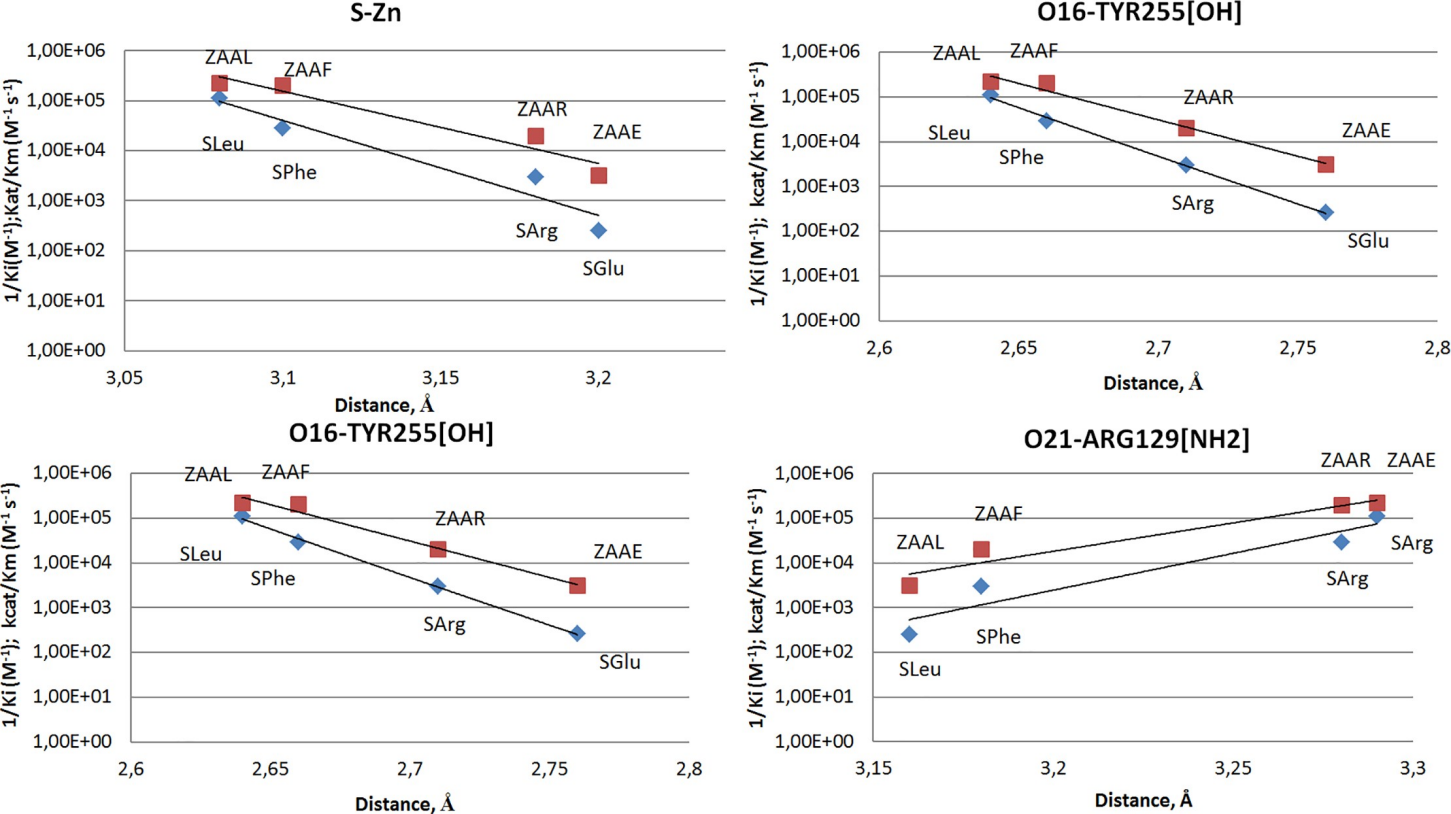
The inhibition of CPT and its catalytic efficiency depend on the gap between the inhibitor and the residues of the enzyme's catalytic center. Association constants (1/K_I_) of the transition state analogs are shown as blue diamonds, catalytic efficiencies (k_cat_/K_M_) on the corresponding substrates are shown as red squares.

In other words, the location of the bound transition state analogue in the active center of the enzyme, the distance and the orientation with respect to the functional groups of the catalytic amino acid residues of the enzyme depend on the nature of its side chain. All these facts allow us to conclude that in the case of CPT catalysis, the interactions of a side chain of the substrate or an analogue of a transition state in the leaving group binding subsite (S1’-subsite) are important not only for effective binding to the enzyme, but also for orientation of the cleavable bond to the catalytic groups to ensure optimal geometry of the transition complex for subsequent covalent conversion, which primarily relates to the lengths of ionic and hydrogen bonds. This is also indicated by the correlation between the binding constants of CPT with the transition state analogues (sulfamoyl inhibitors) and the distance between the sulfur atom and zinc ion: the inhibition constant and bond length increase in the same sequence SLeu < SPhe < SArg < SGlu (Table 4). The inhibition constants of the CPT by different sulfamide inhibitors also correlates with the hydrogen bond length between the carboxyl groups of Glu277 and O20 oxygen atom of the sulfamoyl group, and the hydrogen bond length between the O16 oxygen of the sulfamoyl group and the Tyr255 hydroxyl group (Fig 2, Table 3). Depending on the lengths of these bonds, not only the inhibition constant by the transition state analogues changes, but also the efficiency of the catalytic conversion of the corresponding substrates (Table 4), indicating the adequacy of structural modelling of the transition states in the CPT-catalyzed reactions by using sulfamoyl derivatives of amino acids.

**Table 4:** Catalysis/inhibition parameters for CPT.

Ligand, parameter, unit	Value	SD
SPhe, K_I_, M	3.51×10^−5^	4.22×10^−6^
ZAAF, k_cat_/K_m_, M^-1^s^-1^	2.0×10^5^ [17]	-
SLeu, K_I_, M	8.94×10^−6^	1.23×10^−6^
ZAAL, k_cat_/K_m_, M^-1^s^-1^	2.2×10^5^	5.88×10^4^
SArg, K_I_, M	3.4×10^−4^	8.8×10^−6^
ZAAR, k_cat_/K_m_, M^-1^s^-1^	1.95×10^4^	4.5×10^3^
SGlu, K_I_, M	3.09×10^−3^	5.56×10^−4^
ZAAE, k_cat_/K_m_, M^-1^s^-1^	3.17×10^3^	1.84×10^3^

SD—standard deviation

When comparing the crystal structures of CPT complexes with the sulfamoyl transition state analogues SArg, SPhe, SLeu, or SGlu, it has been observed that sulfur atoms are placed along the axis common to all ligand molecules. At the same time, hydrophobic inhibitors are somewhat displaced relative to hydrophilic ones (Fig 1). The different shift of the ligands, on the other hand, is caused by the different binding of their side chains in the S1’-subsite.

**Fig 3:**
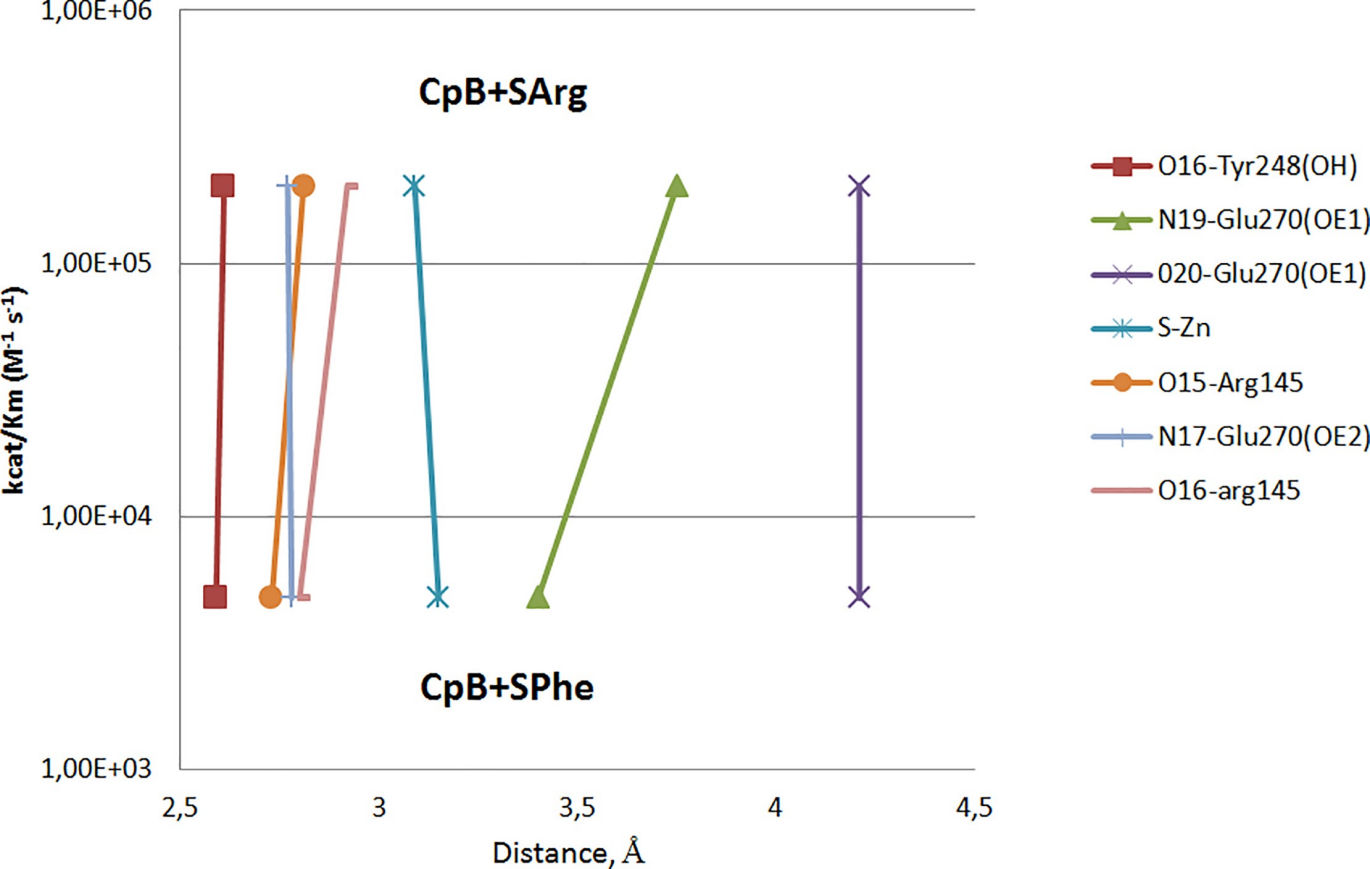
There are virtually no differences in geometry of the CPB complexes with the transition state analogs of different nature (SPhe and SArg). The kinetic parameters were obtained using tripeptide substrates ZAAL and ZAAR.

A different situation was observed in case of CPB, a homologous enzyme with narrow substrate specificity. When moving from the SPhe complex [41] to the SArg complex [40], the Zn-S distance does not change (Fig 3). However, due to the rotation of the ligand around its axis, the side chain carboxyl of Glu270 is shifted by 0.28 Å, the C-terminal oxygen atom by 0.26 Å, and the phenol hydroxyl of Tyr248 by 0.32 Å. This can also have an effect on the efficiency of positively charged and hydrophobic substrates conversion by CPB.

## Conclusions

The increasing CPT affinity to the transition state analogs in the order of SGlu, SArg, SPhe, SLeu (the inhibition constants 3.9×10^−3^> 3.4×10^−4^> 3.5×10^−5^ > 8.9×10^−6^ M) correlates well with a decreasing Zn-S gap in these complexes (3.2 > 3.18 > 3.1 > 3.08 Å) and the increasing efficiency of CPT-catalyzed hydrolysis of the corresponding tripeptide substrates ZAAL > ZAAF > ZAAR > ZAAE. Thus, the side chain of the substrates’ leaving group affects the efficiency of CPT catalysis by governing the orientation of the cleavable bond toward the catalytic residues, regulating small changes of the corresponding distances and defining the structure of the transition complex. This is due to a subtle structural organization and functional role of the S1'-subsite of CPT that maintains the broad substrate specificity of this enzyme in contrast to other homologous metallocarboxypeptidases with predominantly narrow substrate profiles.

## Supporting information

S1 File Kinetic data for K_I_ determination. (PDF)

S2 File Structure validation report for 6GO2 PDB entry. (PDF)

S3 File Structure validation report for 6SN6 PDB entry. (PDF)
