# Supplementary material for: The nature of the ligand’s side chain interacting with the S1'-subsite of metallocarboxypeptidase T (from Thermoactinomyces vulgaris) determines the geometry of the tetrahedral transition complex
Source: PLoS One. 2019 Dec 30;14(12):e0226636. doi: 10.1371/journal.pone.0226636 (PMC6937156; doi:10.1371/journal.pone.0226636)
Supplement: S1 File — (PDF) [file pone.0226636.s001.pdf]

Supplement 1. The progress curves of the CPT-catalyzed conversion of the tripeptide substrate (ZAAL) obtained in the presence of different sulfamoyl inhibitors.

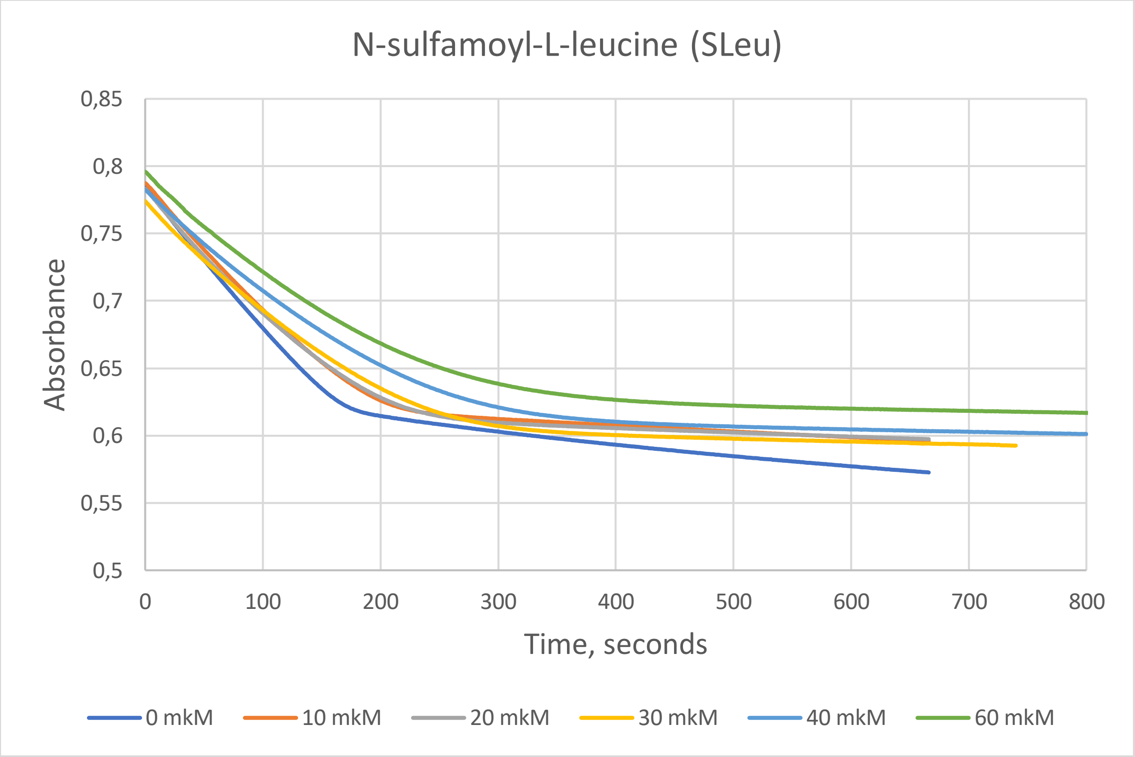

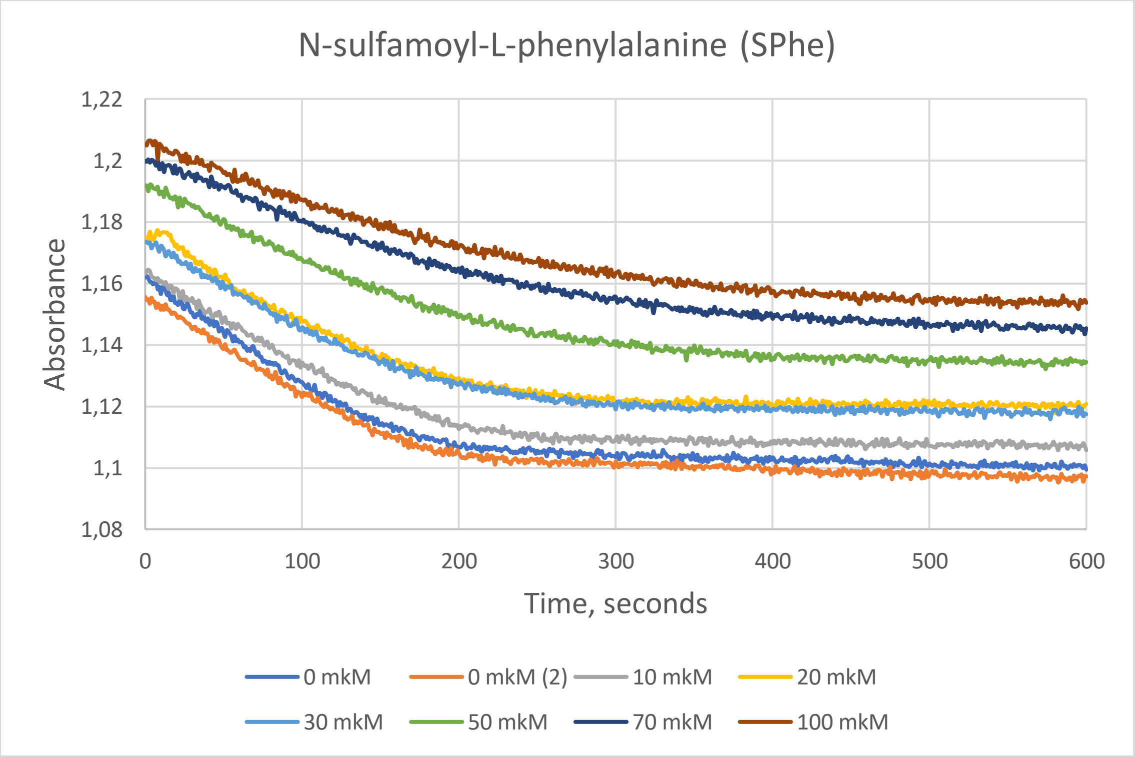

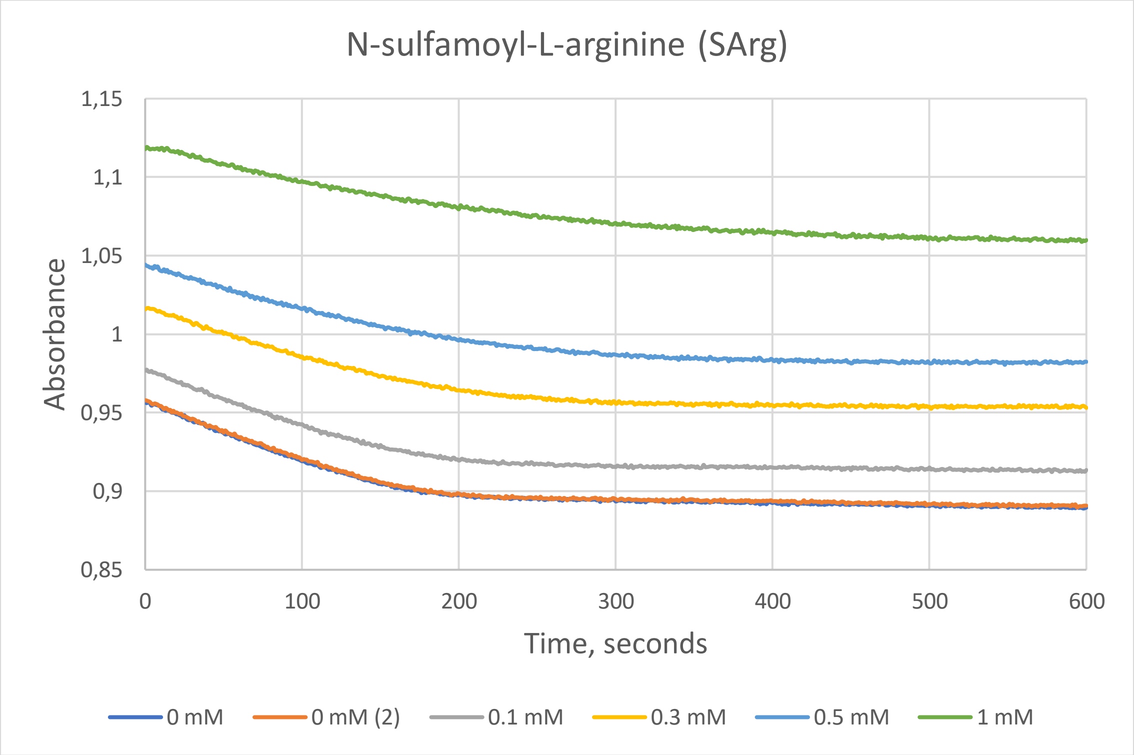

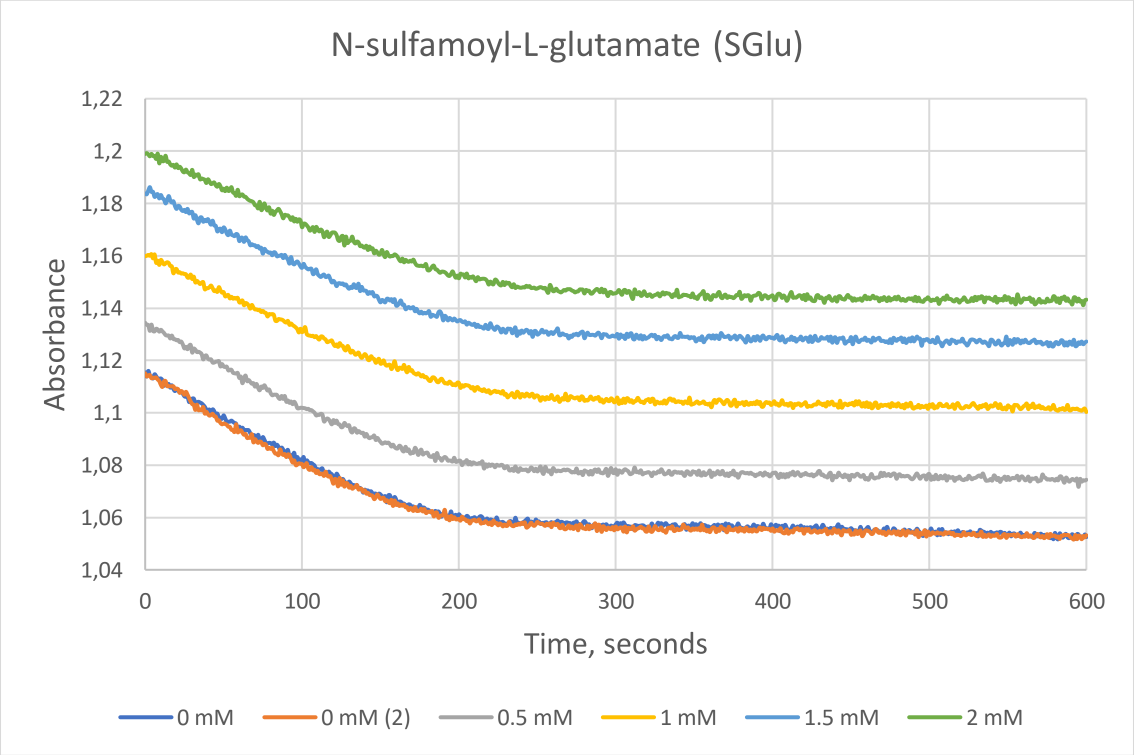

The kinetic experiment conditions: 25 °C, 0.25 M Tris-HCl buffer solution with pH 7.5 containing 10 mM $CaCl_2$. The light absorbance was measured at 225 nm. The tripeptide substrate (ZAAL) concentration: 0.2 mM (SPhe, SArg, SGlu) or 0.5 mM (SLeu). The enzyme (wild type carboxypeptidase T) concentration: 55 nM (SPhe, SArg, SGlu) or 275 nM (SLeu).

Supplement 2. The apparent K_M/V_{max} value of the tripeptide substrate (ZAAL) conversion by the wild type carboxypeptidase T vs sulfamoyl inhibitor concentration graphs.

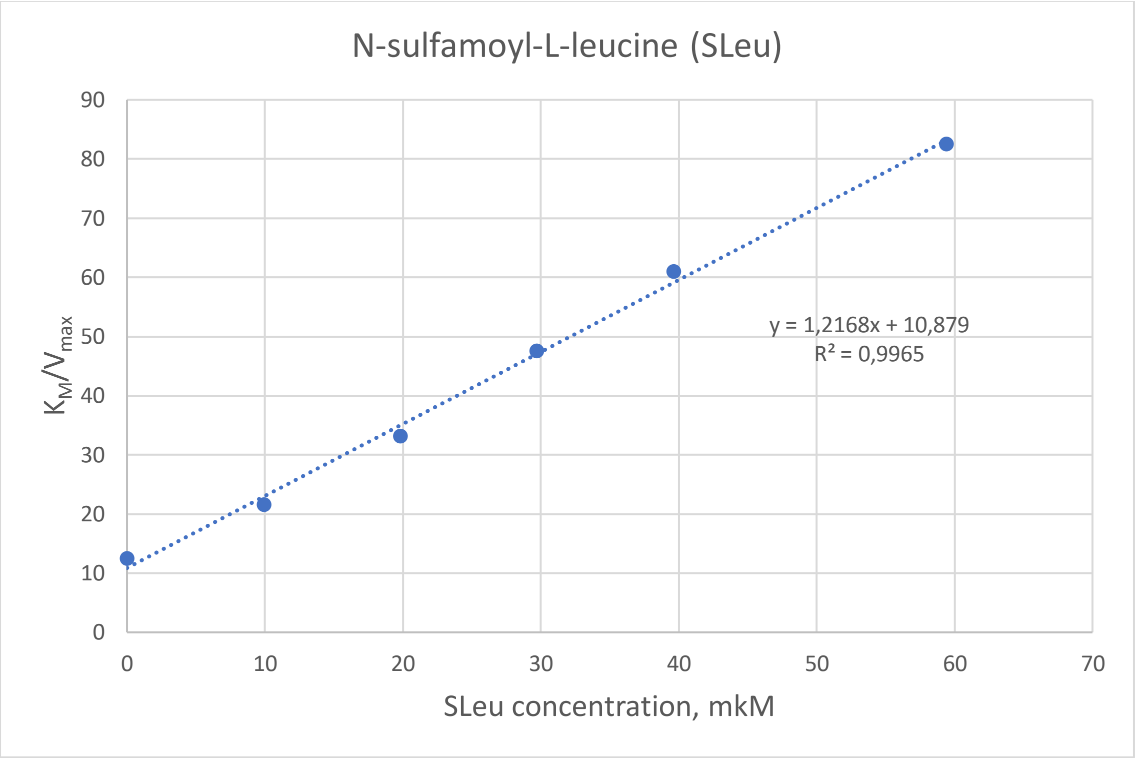

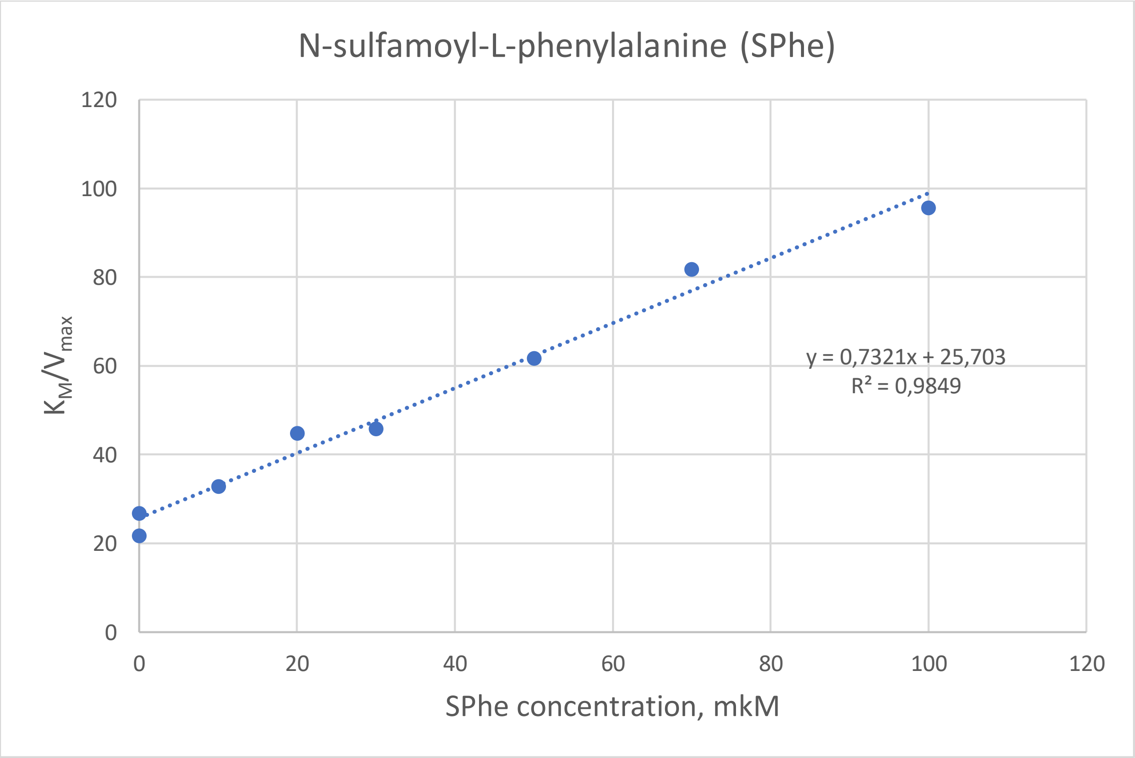

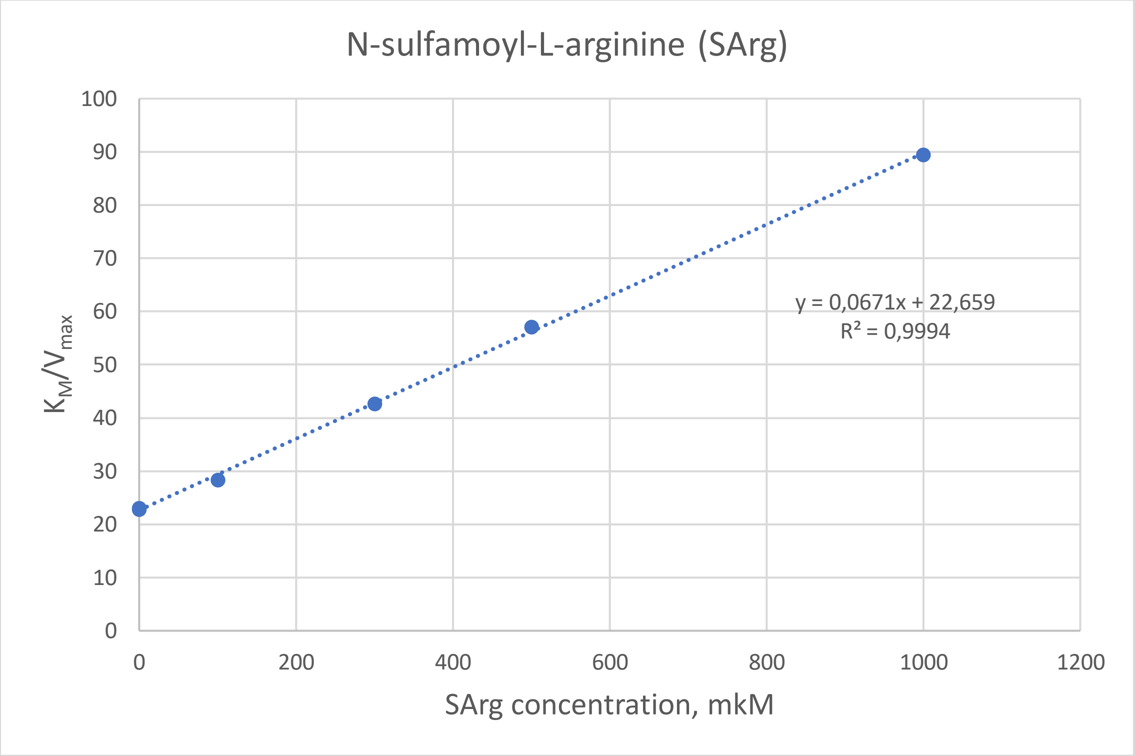

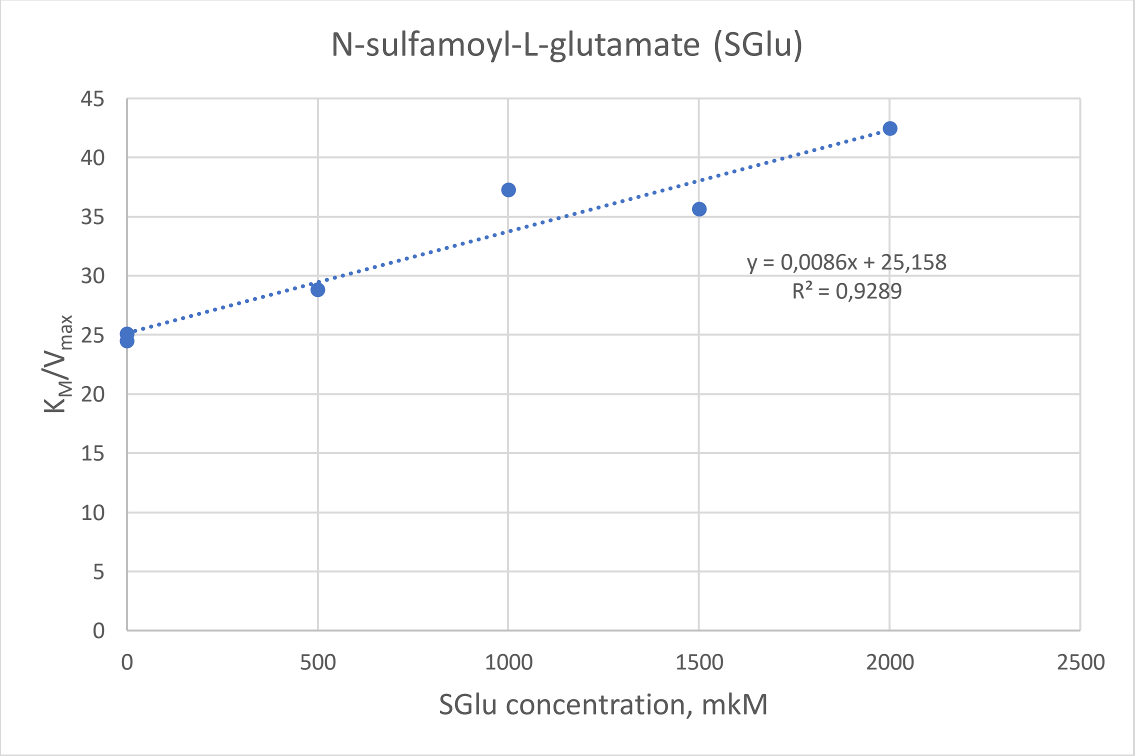

The final K_I value was found as the approximating line's y-intercept divided by the slope.
