# Supplementary material for: The nature of the ligand’s side chain interacting with the S1'-subsite of metallocarboxypeptidase T (from Thermoactinomyces vulgaris) determines the geometry of the tetrahedral transition complex
Source: PLoS One. 2019 Dec 30;14(12):e0226636. doi: 10.1371/journal.pone.0226636 (PMC6937156; doi:10.1371/journal.pone.0226636)
Supplement: S3 File — (PDF) [file pone.0226636.s003.pdf]

Full wwPDB X-ray Structure Validation Report (i)

Aug 23, 2019 – 03:12 pm BST

PDB ID	:	6SN6
Title	:	CARBOXYPEPTIDASE T WITH N-SULFAMOYL-L-GLUTAMIC ACID
Deposited on	:	2019-08-23
Resolution	:	1.93 Å(reported)

This is a Full wwPDB X-ray Structure Validation Report.

This report is produced by the wwPDB biocuration pipeline after annotation of the structure.

We welcome your comments at *validation@mail.wwpdb.org*
A user guide is available at
https://www.wwpdb.org/validation/2017/XrayValidationReportHelp
with specific help available everywhere you see the (i) symbol.

CONFIDENTIAL VALIDATION REPORT

The following versions of software and data (see references (i)) were used in the production of this report:

MolProbity	:	4.02b-467
Xtriage (Phenix)	:	1.13
EDS	:	2.4
buster-report	:	1.1.7 (2018)
Percentile statistics	:	20171227.v01 (using entries in the PDB archive December 27th 2017)
Refmac	:	5.8.0158
CCP4	:	7.0 (Gargrove)
Ideal geometry (proteins)	:	Engh & Huber (2001)
Ideal geometry (DNA, RNA)	:	Parkinson et al. (1996)
Validation Pipeline (wwPDB-VP)	:	2.4

1 Overall quality at a glance (i)

The following experimental techniques were used to determine the structure:
X-RAY DIFFRACTION

The reported resolution of this entry is 1.93 Å.

Percentile scores (ranging between 0-100) for global validation metrics of the entry are shown in the following graphic. The table shows the number of entries on which the scores are based.

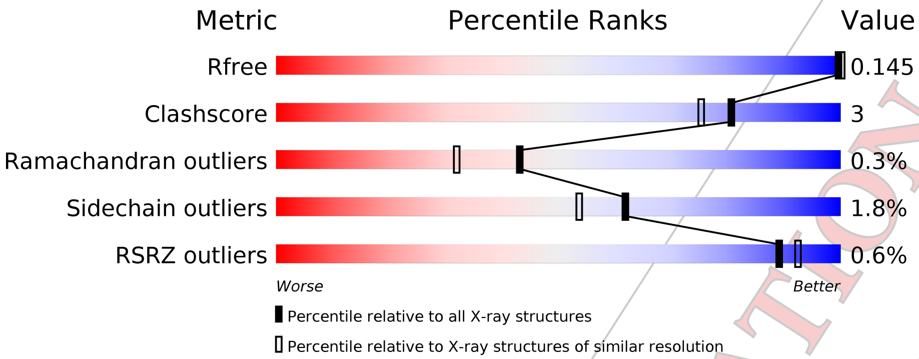

Metric	Whole archive (#Entries)	Similar resolution (#Entries, resolution range(Å))
R_{free}	111664	3622 (1.96-1.92)
Clashscore	122126	3795 (1.96-1.92)
Ramachandran outliers	120053	3757 (1.96-1.92)
Sidechain outliers	120020	3757 (1.96-1.92)
RSRZ outliers	108989	3554 (1.96-1.92)

The table below summarises the geometric issues observed across the polymeric chains and their fit to the electron density. The red, orange, yellow and green segments on the lower bar indicate the fraction of residues that contain outliers for >=3, 2, 1 and 0 types of geometric quality criteria. A grey segment represents the fraction of residues that are not modelled. The numeric value for each fraction is indicated below the corresponding segment, with a dot representing fractions <=5% The upper red bar (where present) indicates the fraction of residues that have poor fit to the electron density. The numeric value is given above the bar.

Mol	Chain	Length	Quality of chain
1	A	323	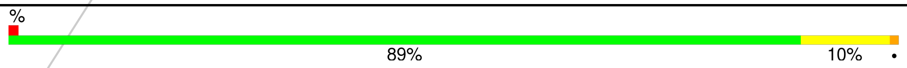

CONFIDENTIAL VALIDATION REPORT

2 Entry composition (i)

There are 5 unique types of molecules in this entry. The entry contains 3018 atoms, of which 0 are hydrogens and 0 are deuteriums.

In the tables below, the ZeroOcc column contains the number of atoms modelled with zero occupancy, the AltConf column contains the number of residues with at least one atom in alternate conformation and the Trace column contains the number of residues modelled with at most 2 atoms.

- Molecule 1 is a protein called Carboxypeptidase T.

Mol	Chain	Residues	Atoms					ZeroOcc	AltConf	Trace
			Total	C	N	O	S			
1	A	323	2678	1679	445	542	12	0	14	0

- Molecule 2 is ZINC ION (three-letter code: ZN) (formula: Zn).

Mol	Chain	Residues	Atoms		ZeroOcc	AltConf
			Total	Zn		
2	A	1	1	1	0	0

- Molecule 3 is N-sulfamoyl-L-glutamic acid (three-letter code: 3K0) (formula: $C_5H_{10}N_2O_6S$) (labeled as "Ligand of Interest" by author).

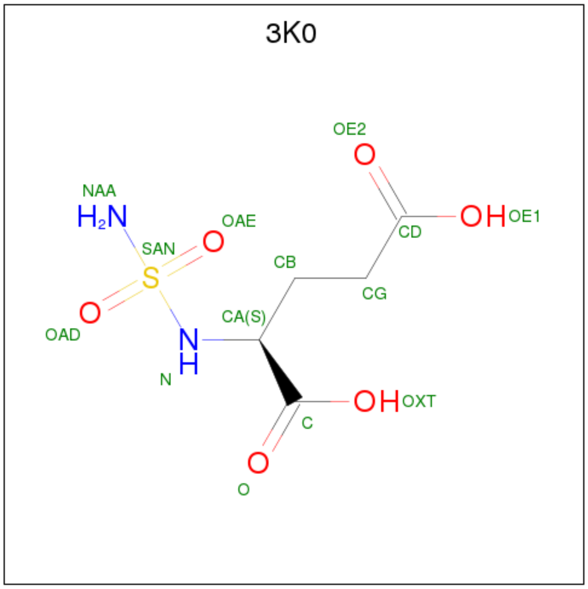

Mol	Chain	Residues	Atoms					ZeroOcc	AltConf
			Total	C	N	O	S		
3	A	1	14	5	2	6	1	0	0

CONFIDENTIAL VALIDATION REPORT

- Molecule 4 is CALCIUM ION (three-letter code: CA) (formula: Ca).

Mol	Chain	Residues	Atoms		ZeroOcc	AltConf
			Total	Ca		
4	A	3	3	3	0	0

- Molecule 5 is water.

Mol	Chain	Residues	Atoms		ZeroOcc	AltConf
			Total	O		
5	A	322	322	322	0	0

CONFIDENTIAL VALIDATION REPORT

3 Residue-property plots (i)

These plots are drawn for all protein, RNA and DNA chains in the entry. The first graphic for a chain summarises the proportions of the various outlier classes displayed in the second graphic. The second graphic shows the sequence view annotated by issues in geometryand electron density. Residues are color-coded according to the number of geometric quality criteria for which they contain at least one outlier: green = 0, yellow = 1, orange = 2 and red = 3 or more. A red dot above a residue indicates a poor fit to the electron density (RSRZ > 2). Stretches of 2 or more consecutive residues without any outlier are shown as a green connector. Residues present in the sample, but not in the model, are shown in grey.

- Molecule 1: Carboxypeptidase T

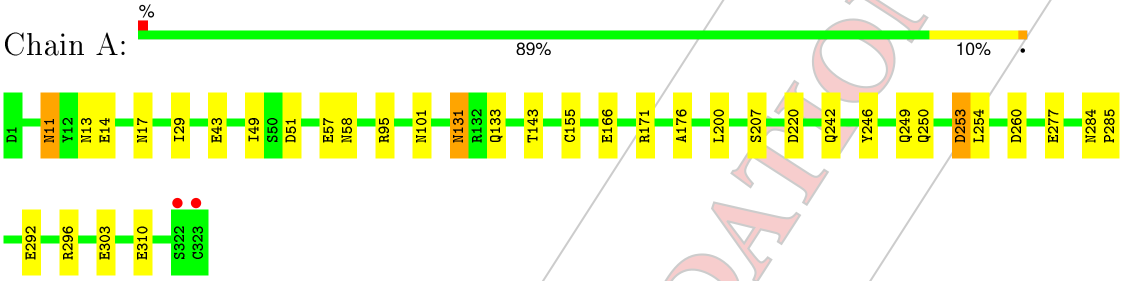

CONFIDENTIAL VALIDATION REPORT

4 Data and refinement statistics (i)

Property	Value	Source
Space group	P 63 2 2	Depositor
Cell constants<br>a, b, c, α, β, γ	158.73Å 158.73Å 105.67Å<br>90.00° 90.00° 120.00°	Depositor
Resolution (Å)	27.93 – 1.93<br>27.93 – 1.93	Depositor<br>EDS
% Data completeness<br>(in resolution range)	98.9 (27.93-1.93)<br>98.9 (27.93-1.93)	Depositor<br>EDS
R_{merge}	0.14	Depositor
R_{sym}	(Not available)	Depositor
$< I/\sigma(I) >$ [1]	5.38 (at 1.93Å)	Xtriage
Refinement program	REFMAC 5.8.0238	Depositor
R, R_{free}	0.112 , 0.145<br>0.113 , 0.145	Depositor<br>DCC
R_{free} test set	2959 reflections (5.06%)	wwPDB-VP
Wilson B-factor (Å^2)	16.8	Xtriage
Anisotropy	0.063	Xtriage
Bulk solvent k_{sol}(e/Å^3), B_{sol}(Å^2)	0.36 , 45.2	EDS
L-test for twinning[2]	$< \|L\| > = 0.48$, $< L^2 > = 0.31$	Xtriage
Estimated twinning fraction	No twinning to report.	Xtriage
F_o,F_c correlation	0.97	EDS
Total number of atoms	3018	wwPDB-VP
Average B, all atoms (Å^2)	19.0	wwPDB-VP

Xtriage's analysis on translational NCS is as follows: *The largest off-origin peak in the Patterson function is 2.67% of the height of the origin peak. No significant pseudotranslation is detected.*

[1]Intensities estimated from amplitudes.

[2]Theoretical values of $< |L| >$, $< L^2 >$ for acentric reflections are 0.5, 0.333 respectively for untwinned datasets, and 0.375, 0.2 for perfectly twinned datasets.

CONFIDENTIAL VALIDATION REPORT

5 Model quality (i)

5.1 Standard geometry (i)

Bond lengths and bond angles in the following residue types are not validated in this section: ZN, CA, 3K0

The Z score for a bond length (or angle) is the number of standard deviations the observed value is removed from the expected value. A bond length (or angle) with $|Z| > 5$ is considered an outlier worth inspection. RMSZ is the root-mean-square of all Z scores of the bond lengths (or angles).

Mol	Chain	Bond lengths		Bond angles	
		RMSZ	#|Z| >5	RMSZ	#|Z| >5
1	A	1.02	11/2747 (0.4%)	0.98	2/3735 (0.1%)

Chiral center outliers are detected by calculating the chiral volume of a chiral center and verifying if the center is modelled as a planar moiety or with the opposite hand.A planarity outlier is detected by checking planarity of atoms in a peptide group, atoms in a mainchain group or atoms of a sidechain that are expected to be planar.

Mol	Chain	#Chirality outliers	#Planarity outliers
1	A	0	1

All (11) bond length outliers are listed below:

Mol	Chain	Res	Type	Atoms	Z	Observed(Å)	Ideal(Å)
1	A	303	GLU	CD-OE2	8.32	1.34	1.25
1	A	277	GLU	CD-OE2	7.68	1.34	1.25
1	A	57	GLU	CD-OE2	-7.49	1.17	1.25
1	A	292	GLU	CD-OE2	7.12	1.33	1.25
1	A	277	GLU	CD-OE1	6.43	1.32	1.25
1	A	43	GLU	CD-OE2	-6.25	1.18	1.25
1	A	166	GLU	CD-OE1	-5.57	1.19	1.25
1	A	253	ASP	CG-OD1	5.37	1.37	1.25
1	A	303	GLU	CD-OE1	5.26	1.31	1.25
1	A	310	GLU	CD-OE2	5.19	1.31	1.25
1	A	296	ARG	CZ-NH2	-5.01	1.26	1.33

All (2) bond angle outliers are listed below:

Mol	Chain	Res	Type	Atoms	Z	Observed(^{o})	Ideal(^{o})
1	A	95	ARG	NE-CZ-NH1	-7.86	116.37	120.30
1	A	246	TYR	CB-CG-CD1	5.64	124.39	121.00

CONFIDENTIAL VALIDATION REPORT

There are no chirality outliers.

All (1) planarity outliers are listed below:

Mol	Chain	Res	Type	Group
1	A	155	CYS	Peptide

5.2 Too-close contacts (i)

In the following table, the Non-H and H(model) columns list the number of non-hydrogen atoms and hydrogen atoms in the chain respectively. The H(added) column lists the number of hydrogen atoms added and optimized by MolProbity. The Clashes column lists the number of clashes within the asymmetric unit, whereas Symm-Clashes lists symmetry related clashes.

Mol	Chain	Non-H	H(model)	H(added)	Clashes	Symm-Clashes
1	A	2678	0	2483	14	1
2	A	1	0	0	0	0
3	A	14	0	8	1	0
4	A	3	0	0	0	0
5	A	322	0	0	3	3
All	All	3018	0	2491	15	4

The all-atom clashscore is defined as the number of clashes found per 1000 atoms (including hydrogen atoms). The all-atom clashscore for this structure is 3.

All (15) close contacts within the same asymmetric unit are listed below, sorted by their clash magnitude.

Atom-1	Atom-2	Interatomic distance (Å)	Clash overlap (Å)
1:A:11:ASN:HD22	1:A:11:ASN:C	1.97	0.68
1:A:220:ASP:HA	1:A:250:GLN:HE22	1.61	0.65
1:A:51:ASP:HB3	1:A:101:ASN:HD22	1.64	0.61
1:A:131:ASN:ND2	1:A:143:THR:H	2.00	0.59
1:A:11:ASN:ND2	1:A:14:GLU:H	2.00	0.57
1:A:133:GLN:HE22	1:A:176:ALA:HA	1.73	0.54
1:A:29:ILE:O	1:A:49:ILE:HA	2.12	0.49
1:A:200[B]:LEU:HD12	1:A:200[B]:LEU:C	2.34	0.47
1:A:171:ARG:NH2	5:A:511:HOH:O	2.51	0.43
1:A:13:ASN:ND2	5:A:504:HOH:O	2.36	0.43
3:A:402:3K0:OAE	3:A:402:3K0:C	2.67	0.42
1:A:284[B]:ASN:HA	1:A:285:PRO:HA	1.89	0.41
1:A:51:ASP:HB3	1:A:101:ASN:ND2	2.30	0.41
1:A:249[A]:GLN:HG3	1:A:253:ASP:HB2	2.04	0.40
1:A:17[B]:ASN:ND2	5:A:506:HOH:O	2.37	0.40

CONFIDENTIAL VALIDATION REPORT

All (4) symmetry-related close contacts are listed below. The label for Atom-2 includes the symmetry operator and encoded unit-cell translations to be applied.

Atom-1	Atom-2	Interatomic distance (Å)	Clash overlap (Å)
1:A:242:GLN:OE1	1:A:284[B]:ASN:OD1[11_654]	1.38	0.82
5:A:685:HOH:O	5:A:685:HOH:O[8_555]	1.83	0.37
5:A:678:HOH:O	5:A:678:HOH:O[10_554]	1.95	0.25
5:A:552:HOH:O	5:A:685:HOH:O[8_555]	1.99	0.21

5.3 Torsion angles (i)

5.3.1 Protein backbone (i)

In the following table, the Percentiles column shows the percent Ramachandran outliers of the chain as a percentile score with respect to all X-ray entries followed by that with respect to entries of similar resolution.

The Analysed column shows the number of residues for which the backbone conformation was analysed, and the total number of residues.

Mol	Chain	Analysed	Favoured	Allowed	Outliers	Percentiles	
1	A	335/323 (104%)	327 (98%)	7 (2%)	1 (0%)	43	32

All (1) Ramachandran outliers are listed below:

Mol	Chain	Res	Type
1	A	207	SER

5.3.2 Protein sidechains (i)

In the following table, the Percentiles column shows the percent sidechain outliers of the chain as a percentile score with respect to all X-ray entries followed by that with respect to entries of similar resolution.

The Analysed column shows the number of residues for which the sidechain conformation was analysed, and the total number of residues.

Mol	Chain	Analysed	Rotameric	Outliers	Percentiles	
1	A	296/282 (105%)	291 (98%)	5 (2%)	63	56

All (5) residues with a non-rotameric sidechain are listed below:

CONFIDENTIAL VALIDATION REPORT

Mol	Chain	Res	Type
1	A	11	ASN
1	A	58	ASN
1	A	131	ASN
1	A	254	LEU
1	A	260	ASP

Some sidechains can be flipped to improve hydrogen bonding and reduce clashes. All (8) such sidechains are listed below:

Mol	Chain	Res	Type
1	A	11	ASN
1	A	58	ASN
1	A	101	ASN
1	A	131	ASN
1	A	133	GLN
1	A	187	ASN
1	A	250	GLN
1	A	268	GLN

5.3.3 RNA (i)

There are no RNA molecules in this entry.

5.4 Non-standard residues in protein, DNA, RNA chains (i)

There are no non-standard protein/DNA/RNA residues in this entry.

5.5 Carbohydrates (i)

There are no carbohydrates in this entry.

5.6 Ligand geometry (i)

Of 5 ligands modelled in this entry, 4 are monoatomic - leaving 1 for Mogul analysis.

There are no bond length outliers.

There are no bond angle outliers.

There are no chirality outliers.

There are no torsion outliers.

CONFIDENTIAL VALIDATION REPORT

There are no ring outliers.

No monomer is involved in short contacts.

The following is a two-dimensional graphical depiction of Mogul quality analysis of bond lengths, bond angles, torsion angles, and ring geometry for all instances of the Ligand of Interest. In addition, ligands with molecular weight > 250 and outliers as shown on the validation Tables will also be included. For torsion angles, if less then 5% of the Mogul distribution of torsion angles is within 10 degrees of the torsion angle in question, then that torsion angle is considered an outlier. Any bond that is central to one or more torsion angles identified as an outlier by Mogul will be highlighted in the graph. For rings, the root-mean-square deviation (RMSD) between the ring in question and similar rings identified by Mogul is calculated over all ring torsion angles. If the average RMSD is greater than 60 degrees and the minimal RMSD between the ring in question and any Mogul-identified rings is also greater than 60 degrees, then that ring is considered an outlier. The outliers are highlighted in purple. The color gray indicates Mogul did not find sufficient equivalents in the CSD to analyse the geometry.

**Ligand 3K0 A 402**	
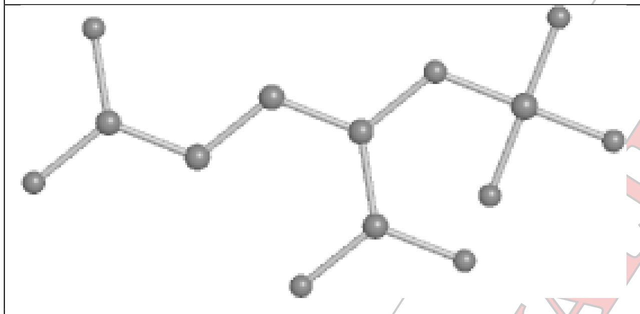 **Bond lengths**	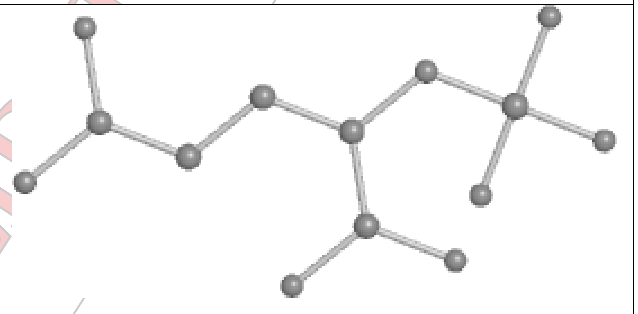 **Bond angles**
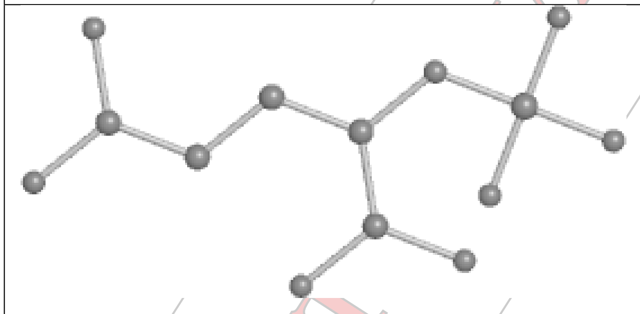 **Torsions**	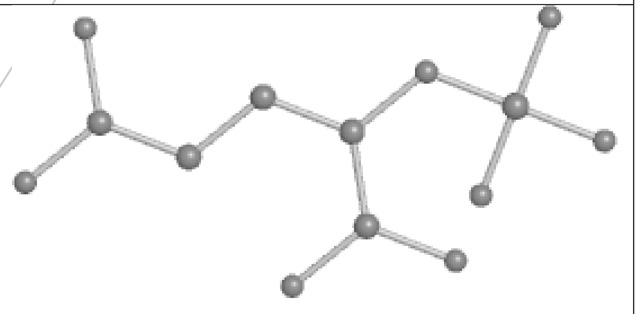 **Rings**

5.7 Other polymers (i)

There are no such residues in this entry.

5.8 Polymer linkage issues (i)

There are no chain breaks in this entry.

CONFIDENTIAL VALIDATION REPORT

6 Fit of model and data (i)

6.1 Protein, DNA and RNA chains (i)

In the following table, the column labelled '#RSRZ> 2' contains the number (and percentage) of RSRZ outliers, followed by percent RSRZ outliers for the chain as percentile scores relative to all X-ray entries and entries of similar resolution. The OWAB column contains the minimum, median, 95th percentile and maximum values of the occupancy-weighted average B-factor per residue. The column labelled 'Q< 0.9' lists the number of (and percentage) of residues with an average occupancy less than 0.9.

Mol	Chain	Analysed	<RSRZ>	#RSRZ>2			OWAB(Å^2)	Q<0.9
1	A	323/323 (100%)	-0.76	2 (0%)	89	92	11, 16, 29, 58	0

All (2) RSRZ outliers are listed below:

Mol	Chain	Res	Type	RSRZ
1	A	323	CYS	2.9
1	A	322	SER	2.6

6.2 Non-standard residues in protein, DNA, RNA chains (i)

There are no non-standard protein/DNA/RNA residues in this entry.

6.3 Carbohydrates (i)

There are no carbohydrates in this entry.

6.4 Ligands (i)

In the following table, the Atoms column lists the number of modelled atoms in the group and the number defined in the chemical component dictionary. The B-factors column lists the minimum, median, 95th percentile and maximum values of B factors of atoms in the group. The column labelled 'Q< 0.9' lists the number of atoms with occupancy less than 0.9.

Mol	Type	Chain	Res	Atoms	RSCC	RSR	B-factors(Å^2)	Q<0.9
3	3K0	A	402	14/14	0.96	0.10	17,27,64,68	0
2	ZN	A	401	1/1	1.00	0.04	13,13,13,13	0
4	CA	A	404	1/1	1.00	0.05	23,23,23,23	0
4	CA	A	403	1/1	1.00	0.03	19,19,19,19	0
4	CA	A	405	1/1	1.00	0.04	25,25,25,25	0

CONFIDENTIAL VALIDATION REPORT

The following is a graphical depiction of the model fit to experimental electron density of all instances of the Ligand of Interest. In addition, ligands with molecular weight > 250 and outliers as shown on the geometry validation Tables will also be included. Each fit is shown from different orientation to approximate a three-dimensional view.

Electron density around 3K0 A 402:

$2mF_o$-DF_c (at 0.7 rmsd) in gray
mF_o-DF_c (at 3 rmsd) in purple (negative)
and green (positive)

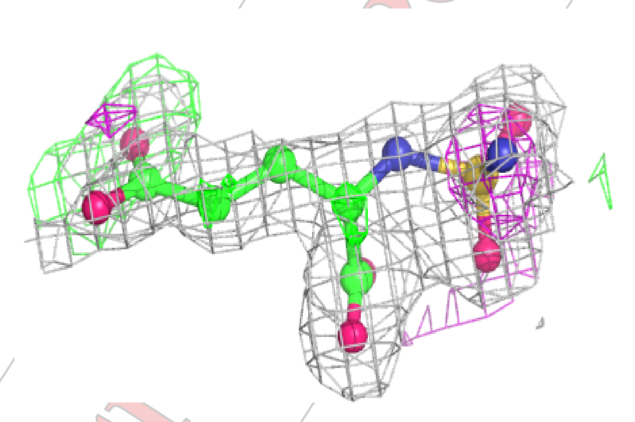

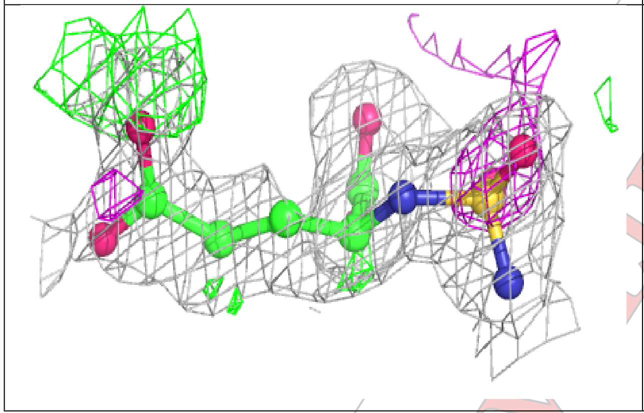

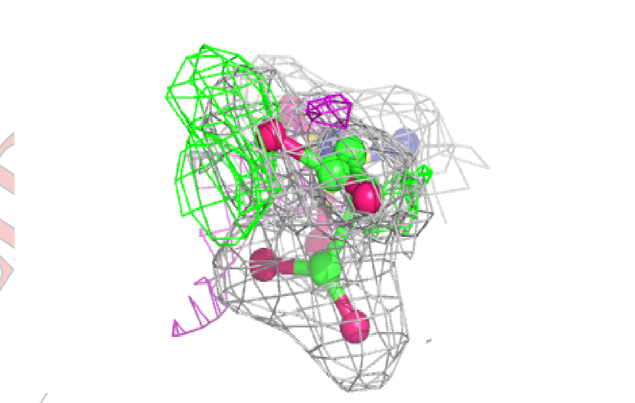

6.5 Other polymers ⓘ

There are no such residues in this entry.

CONFIDENTIAL
